# Overview of Bat and Wildlife Coronavirus Surveillance in Africa: A Framework for Global Investigations

**DOI:** 10.3390/v13050936

**Published:** 2021-05-18

**Authors:** Marike Geldenhuys, Marinda Mortlock, Jonathan H. Epstein, Janusz T. Pawęska, Jacqueline Weyer, Wanda Markotter

**Affiliations:** 1 Centre for Viral Zoonoses, Department of Medical Virology, Faculty of Health Sciences, University of Pretoria, Pretoria 0001, South Africa; marike.geldenhuys@up.ac.za (M.G.); marinda.mortlock@up.ac.za (M.M.); epstein@ecohealthalliance.org (J.H.E.); januszp@nicd.ac.za (J.T.P.); jacquelinew@nicd.ac.za (J.W.); 2 EcoHealth Alliance, New York, NY 10001, USA; 3 Centre for Emerging Zoonotic and Parasitic Diseases, National Institute for Communicable Diseases, Johannesburg 2131, South Africa; 4 Department of Microbiology and Infectious Diseases, School of Pathology, University of Witwatersrand, Johannesburg 2131, South Africa

**Keywords:** coronaviruses, surveillance, biosurveillance, Africa, bat, emerging, African bat coronaviruses, wildlife, domestic animals, COVID-19, HCoV-229E, HCoV-NL63, MERS-CoV, SARS-CoV, SARS-CoV 2, surveillance strategies

## Abstract

The ongoing coronavirus disease 2019 (COVID-19) pandemic has had devastating health and socio-economic impacts. Human activities, especially at the wildlife interphase, are at the core of forces driving the emergence of new viral agents. Global surveillance activities have identified bats as the natural hosts of diverse coronaviruses, with other domestic and wildlife animal species possibly acting as intermediate or spillover hosts. The African continent is confronted by several factors that challenge prevention and response to novel disease emergences, such as high species diversity, inadequate health systems, and drastic social and ecosystem changes. We reviewed published animal coronavirus surveillance studies conducted in Africa, specifically summarizing surveillance approaches, species numbers tested, and findings. Far more surveillance has been initiated among bat populations than other wildlife and domestic animals, with nearly 26,000 bat individuals tested. Though coronaviruses have been identified from approximately 7% of the total bats tested, surveillance among other animals identified coronaviruses in less than 1%. In addition to a large undescribed diversity, sequences related to four of the seven human coronaviruses have been reported from African bats. The review highlights research gaps and the disparity in surveillance efforts between different animal groups (particularly potential spillover hosts) and concludes with proposed strategies for improved future biosurveillance.

## 1. Introduction

In the past two decades, four novel coronaviruses of public and veterinary health importance have emerged. These include the three agents originating from China; severe acute respiratory syndrome coronavirus (SARS-CoV) in 2002 [1,2], swine acute diarrhea syndrome coronavirus (SADS-CoV) among localized pig farms in 2017 with re-emergence in 2019 [3,4], and SARS-CoV 2 towards the end of 2019 [1,4,5,6]. The fourth emergent coronavirus, Middle East respiratory syndrome coronavirus (MERS-CoV), emerged in the Arabian Peninsula in 2012 [7,8]. These events show that coronaviruses have the potential to spillover from natural hosts into different species and cause severe diseases with devastating consequences. Dromedary camels are considered the reservoirs of MERS-CoV, though the original source and transmission routes from animals are still uncertain for SARS-CoV and SARS-CoV 2 [9,10,11,12,13], with related viruses identified in bats. Different amplification hosts are considered to be involved in all three human coronavirus (HCoV) outbreaks.

The link between bats and emerging coronaviruses was first considered in 2005 following the identification of coronaviruses related to SARS-CoV in specific Asian rhinolophid bat species [14,15,16]. Since then, a high diversity of coronavirus nucleic acids has been detected in bats, several of which are related to coronaviruses infecting human and domestic animals, with hundreds of unclassified sequences pending characterization. The expanding knowledge of coronavirus diversity has additionally allowed for novel insights into their evolutionary history, including linking bats as the ancestors of specific mammalian coronavirus lineages [17,18]. More specifically, bat coronaviruses with genetic similarity to known coronavirus species, such as HCoV229E and HCoVNL63, are suggested to have acted as ancestors of these human viruses from previous spillover events [19]. 

Biosurveillance of wildlife hosts, including bats, are one of the first steps towards understanding how viruses emerge [20,21] and include identifying viral diversity, host species, and distribution ranges. However, several factors have been implicated in spillover events, including genetic, ecologic, epidemiological, and anthropological elements [22]. Unless the underlying factors are also identified and mitigated, coronaviruses are likely to continue to emerge in the future. 

The high biodiversity on the African continent supports viral species richness, which has been correlated with disease hotspot mapping and novel viral diseases that have emerged or re-emerged in Africa to date [22]. Many communities in Africa live in close contact with wildlife, domesticated animals, and livestock. Some surveillance for bat coronaviruses has been performed in Africa. A recent review by Markotter et al. [23] provides a comprehensive summary of potentially zoonotic coronaviruses reported from Africa (relatives of HCoV229E, HCoVNL63, MERS-CoV, and SARS-CoV), focusing on the distribution of the host bat species, and concluding that inferences on zoonotic potential based on the genetic relatedness is limiting. This review focuses in greater detail on the total coronavirus diversity identified among African animal species. We review published literature concerning bat species targeted, sample sizes, viral genetic diversity, and evolutionary links to specific host species. The review was also expanded to include the currently available surveillance data among non-bat wildlife and domesticated livestock as hosts of coronavirus diversity. We highlight surveillance approaches from previous studies, important findings, and gaps in current surveillance and propose a surveillance framework to guide the design of future biosurveillance studies.

## 2. The Importance of Viral Taxonomy

**Figure 1:**
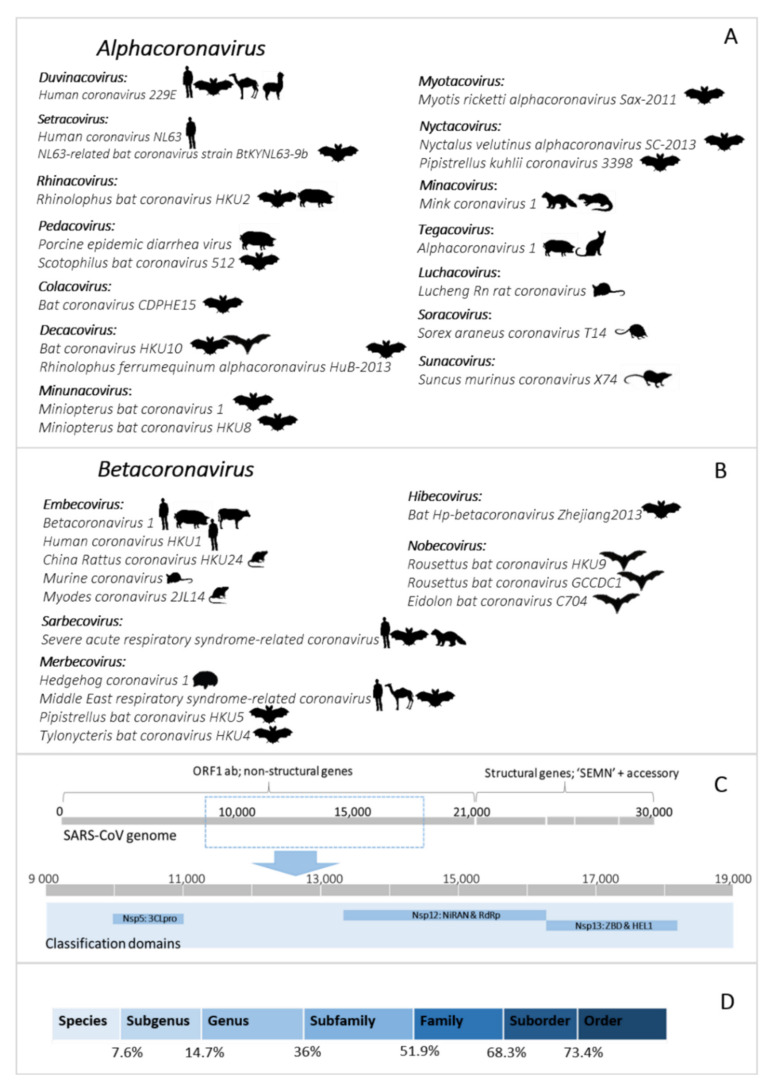
(**A**,**B**) Current coronavirus subgenera (bold) and species of the *Alphacoronavirus* and *Betacoronavirus* genera. The images indicate host species associated with the virus species. Figure constructed with the species listed on the 2019 Release of the ICTV Virus Taxonomy 9th Report MSL#35: (Available at https://talk.ictvonline.org/ictv-reports/ictv_9th_report/positive-sense-rna-viruses-2011/w/posrna_viruses/222/coronaviridae accessed on 12 December 2020). (**C**) Representation of the coronavirus genome (based on the reference genome NC_004718.3 SARS coronavirus Tor2) depicting the locations of important domains for classification of species (NSP5 (3CLpro), NSP12 (NiRAN and RdRp), and NSP13 (ZBD and HEL1)). (**D**) Thresholds of the taxonomic demarcation criteria [24]. Novel viruses are part of a taxonomic level if the divergence within the five concatenated replicase domains is less than the indicated amino acid percentage.

The hierarchical levels of the coronavirus taxonomy are well described [24]. There are currently four genera in the *Orthocoronavirinae* subfamily: the *Alphacoronavirus, Betacoronavirus*, *Gammacoronavirus,* and *Deltacoronavirus*. The *Alphacoronavirus* and *Betacoronavirus* genera predominantly infect mammals and are further divided into subgenera (Figure 1A,B). Human coronaviruses group within either the *Duvinacovirus, Setracovirus, Sarbecovirus, Merbecovirus,* or *Embecovirus* subgenera (Figure 1A,B). Coronavirus genomes consist of several non-structural genes in open reading frame (ORF) 1 (encoding the replicase polyprotein pp1ab), followed by four structural genes and several accessory genes depending on the species (Figure 1C). Current classification criteria for coronaviruses (ICTV code 2019.021S) rely on comparative amino acid sequence analysis of five domains within the replicase polyprotein pp1ab: 3CLpro, NiRAN, RdRp, ZBD, and HEL1 [6,25]. Computational approaches are used to estimate genetic divergence, and thresholds are utilized as demarcation criteria at various taxonomic levels (Figure 1C,D) [24]. Moreover, only complete genomes are considered for formal taxonomic placement.

Since the initial identification of bat coronaviruses in 2005, a total of 16 formally recognized coronavirus species have been described from bats. Biosurveillance research mainly report on partial sequences of the coronavirus genome and can only be described to a limited extent by their phylogenetic grouping or similarity percentages. Sequences are considered ‘related’ to genetically similar sequences in a phylogenetic cluster, pending the viral diversity included in the inference. This ‘related’ terminology has become widely misrepresented. It is frequently used to indicate the relatedness of sequences to the closest human coronavirus (HCoV) in a phylogeny, even if these sequences may be significantly distant. For example, SARS-CoV belongs to the *Sarbecovirus* subgenus; and the *Hibecovirus* subgenus forms a sister-clade to the sarbecoviruses (Figure S2). Sequences with low similarity to sarbecoviruses, and which should be part of the hibecoviruses, have (even recently) been deemed as ‘SARS-related’. Erroneous conclusions may be readily avoided by including all representative diversity of the current taxonomy in phylogenies. In this review, we will employ the convention of limiting the use of ‘related’ only to describe bat coronaviruses deemed sufficiently similar to known species according to demarcation criteria (e.g., MERS-related, SARS-related, 229E-related, and NL63-related). All others will be described in relation to phylogenetic clusters, using sequence similarities where possible, or indicating possible grouping within a subgenus (Figure 1A). 

## 3. Biosurveillance Studies Based on Nucleic Acid Detection in Africa

Table 1 stipulates the selection criteria utilized to identify and classify publications included in the review. Several surveillance studies focused on bat species were identified [19,26,27,28,29,30,31,32,33,34,35,36,37,38,39,40,41,42,43,44,45,46,47], though few studies were found in regards to surveillance among other wild animals or livestock [40,48,49,50,51] (Table 1). This may be due to the ‘reactive’ nature of surveillance among livestock, domestic animals, and non-bat wildlife in response to outbreak events among farmed animals or human populations; such events have not been regularly reported in Africa. Global examples include studies involving farmed civets following the first SARS-CoV outbreak, surveillance in camel herds after identifying MERS-CoV and detecting SARS-CoV 2 among mink farms in Europe [9,52,53]. Coincidentally, the use of passive unbiased metagenomic next-generation sequencing among illegally smuggled pangolins identified sarbecoviruses with overall genome similarity of 85.5% to 92.4% to SARS-CoV 2 in Asia [10,54,55]. 

### 3.1. Surveillance in African Bats

**Figure 2:**
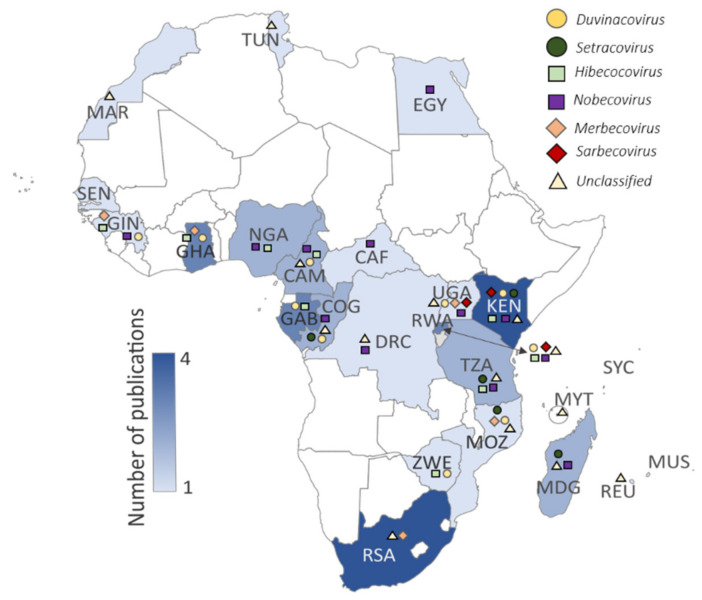
Published bat coronavirus surveillance studies per country (shading denoting the number of publications). Symbols in the key above the map represent different coronaviruses detected in the respective countries: Duvinacovirus as a yellow circle (HCoV229E-related viruses), Setracoronavirus as a dark green circle (HCoVNL63-related viruses), Sarbecoviruses as a red diamond (HCoV-SARS-related viruses), Merbecoviruses as an orange diamond (HCoV-MERS-related viruses), Nobecoviruses as a purple square, Hibecoviruses as a green square, and unclassified viruses as a black triangle. Further details on coronaviruses identified can be reviewed in Table S4. Three-letter ISO country code abbreviations are shown on the map.

Several surveillance studies focused on bats have been performed in Africa since the first reports in 2009 [26,37]. We identified 23 primary surveillance reports and four subsequent secondary characterization reports [57,58,59,60] (Table 2 and Figure 2) that included sampling in 24/54 African countries (www.un.org, accessed on 6 September 2020). Several reports originate from Kenya, Ghana, Gabon, and South Africa (Table 2, Figure 2), with limited surveillance in Morocco and Tunisia [33]. Most studies focused on one or more sites within a single country (Table S1), though few studies include once-off sampling from multiple African countries [30,33,38,45]. Anthony et al. [30] describe the PREDICT surveillance performed over a 5-year timespan in more than 20 countries, seven of which took place in Africa (with Rwanda surveillance further detailed in Nziza et al. [36]). Furthermore, nine reports identified coronaviruses while conducting broader virological surveillance [29,31,32,34,35,36,39,45], whereas others were coronavirus specific. Supplementary Tables S1–S4 summarize the different reports in terms of approach, species and sample numbers, nucleic-acid detection strategy, and overall findings, including when the information was omitted or not sufficiently described.

#### 3.1.1. Sampling Approaches and Methodologies of Bat Coronavirus Surveillance 

**Figure 3:**
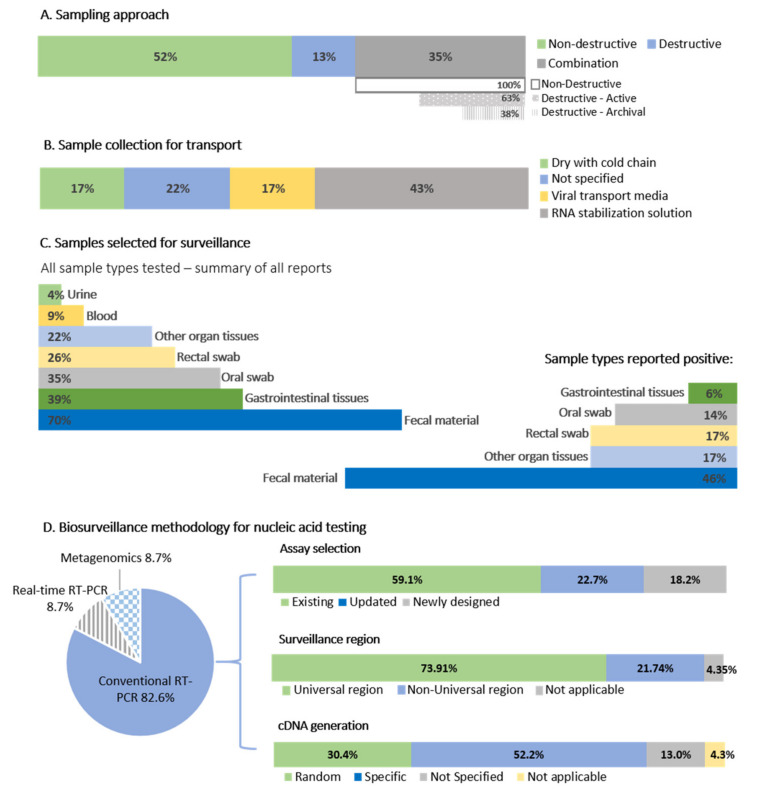
A summary of coronavirus sampling approaches and methodology. (**A**) The sampling approaches of the 23 primary surveillance reports. Combination studies are split into those employing new or archival destructive sampling. (**B**) Sample preservation methods. (**C**) Sample types selected for surveillance and samples testing positive. (**D**) Biosurveillance methodology for nucleic acid testing, percentage of studies using conventional, real-time, or metagenomic approaches. The conventional assays were further split into existing assays from the literature, updated exiting assays, or whether new assays were developed. The percentages of studies targeting the ‘universal surveillance region’ (see text for an explanation) contrast to those using different genome regions, and whether specific or random primers were chosen for cDNA preparation.

Overall, the primary aim of most of the reports was to detect the presence of coronavirus RNA in bat species, with limited subsequent genetic characterization. Bat species and sample numbers were opportunistically sampled at roosts in mainly cross-sectional once-off sampling focused on a targeted population, region, or species. The frequency of sampling was generally poorly described (Table S1). Exceptions include reports from Madagascar, Nigeria, and Zimbabwe, where multiple sampling events (2 or more) were performed at the same roosts [28,31,47]. Figure 3 provides a graphical summary of the approaches employed by surveillance efforts for bat coronaviruses (Tables S1 and S2). 

It is well established that coronaviruses display a gastrointestinal tropism in bats [61], and fecal material or other gastrointestinal sample types such as rectal swabs (non-destructive) or intestinal tissue (destructive) is the preferred sample types for surveillance (Figure 3). Sample collection was mostly non-destructive (52% of studies), including fecal material collected beneath roosting bats in caves and trees [28,29,31,33,37] or fecal material and rectal swabs from individual bats [19,26,27,30,32,34,35,37,42,43,44,46,47]. For this review, we are assuming fecal swabs are the same as rectal swabs. Only 13% of studies solely implemented destructive sampling (collection of organ tissues), and 35% of studies (Figure 3) combined both methodologies to collect sample material for multi-pathogen surveillance [27,30,35,41,43,45] or were tested due to availability within archival tissue banks [32,39,42]. Along with gastrointestinal samples, oral (or throat) swabs were also collected [19,26,27,30,47], but infrequently contained coronavirus RNA [19,27,30,36,39]. Due to limited reporting information provided per study, coronavirus detection among oral swabs can only be roughly estimated. Of all reports investigated, only 35% tested oral swabs (Figure 3). From these reports, 62.5% identified coronavirus RNA, representing positive oral swabs from only 14% of studies overall (Table S1). Coronaviruses were also opportunistically detected within lung and liver tissues [27,38,45], though it is unclear what other positive individuals’ organs were also tested.

**Figure 4:**
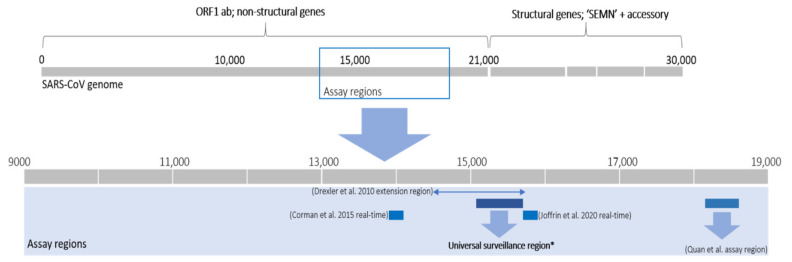
Representation of the coronavirus genome (based on the reference genome NC_004718.3 SARS coronavirus Tor2) depicting the assay regions. The assays corresponding to this universal region included in Tong et al. [26], de Souza Luna [62], Geldenhuys et al. [42] and Geldenhuys et al. [32] (based on primers from Woo et al. [63]), Razanajatovo et al. [47] (based on Poon et al. [14]), Shehata et al. [27], Waruhiu et al. [29] (based on Watanabe et al. [64]), Chu et al. [65], Gouilh et al., [33]. The RdRp grouping units (RGU) amplification region by Drexler et al. [66] is indicated with the line and arrows.

The basic methodology implemented in all but two studies [32,34] involved RNA extraction of samples followed by nucleic acid detection targeting a conserved region of the genome. A region of the RNA dependent RNA polymerase (RdRp) gene within the open reading frame (ORF) 1b of the coronavirus genome (Figure 4) is mostly targeted and corresponds to approximate nucleotide position 15,200–15,600 in the coronavirus genome (using reference NC_004718.3 SARS coronavirus Tor2) (Figure 4, Table S2). Targeting of this “universal coronavirus surveillance region” enables comparison between studies, though 74% of the African bat surveillance studies utilized assays based on the region (22% either used a non-universal region or combination of both; Table S2). The addition of a nested step is generally essential for the detection of low concentration viral RNA. A small number of studies in Africa quantified viral concentrations of positive samples, obtaining as little as 50–450 RNA copies/mg fecal material for some low concentration samples, or between 323 to 1.5 × 10^8^ RNA copies/g of fecal material [37,44].

The majority of surveillance studies (52.2%) implemented a one-step kit approach (i.e., utilizes RNA templates in a single reaction with target-specific primers for cDNA followed directly by PCR amplification), with seven (30.4%) implementing an unbiased methodology for the preparation of cDNA with random hexamers before PCR amplification [31,32,33,35]. An unbiased approach is more beneficial where only limited sample material is available and multi-pathogen surveillance is done. Suitable assays were either selected from the literature (with the assay from de Souza Luna et al. [62] most frequently employed), constitute newly developed assays (included if no reference was provided for assay modifications), or were updated/modified from the literature (Table S2 and Figure 3). Assays selected from the literature were constructed using the available sequence information known at that point in time. The expanding genetic diversity of coronaviruses is high, and even though these assays target a conserved region, existing primers may be less sensitive toward the detection of more diverse viruses. For example, primers developed before the 2012 emergence of MERS-CoV might not be sufficiently sensitive to detect diverse coronaviruses from the Merbecovirus subgenus. Developing new assays or updating available primers have the added advantage of ensuring that some of the expanding sequence diversity of emerging human coronaviruses and newly detected animal coronaviruses can be incorporated; reducing the probability of highly diverse clades going undetected.

Exceptions to this ‘universal CoV surveillance’ region are represented mainly by the nested RT-PCR assay developed by Quan et al. [41], targeting a region downstream of the universal CoV surveillance region, corresponding to the approximate nucleotide position 18,300–18,700 (Figure 4). Sequences amplified with the assay from Quan et al. [41] cannot be directly incorporated in phylogenies using the short universal CoV surveillance region and may only be compared to viruses for which this corresponding genome region is available or with full genomes. The PREDICT surveillance described in Anthony et al. [30] and Nziza et al. [36] utilized two surveillance assays to test samples; that of Watanabe et al. [64] based on the universal region and Quan et al. [41]. In total, the Watanabe assay detected 950 coronavirus sequences compared to the 654 sequences from the Quan assay, with only a 27% overlap [30].

Overall, it is not possible to directly compare methodologies to conclude best practices for coronavirus surveillance. However, non-destructive sampling methodologies (swab collection or fecal material from underneath roosting bats) associated with a gastrointestinal origin allow for successful coronavirus identification with minimal injury to the hosts or ecosystem. Proper preservation of sample material is good practice (cold chain or using preservation media), and unbiased cDNA preparation approaches allow for the conservation of reagents and sample material. The use of appropriate assays and overlapping target regions are essential to enable comparisons between studies.

#### 3.1.2. Summary of Sample Sizes and Bat Species Tested

The surveillance data from the 23 publications were compared to the 2019 African Chiropteran Report (comprehensive report of the current taxonomy with data based on museum records from bats collected across the continent) to determine an estimate of total bats sampled per species (Table S4; [67]). There are 13 extant bat families in Africa, with an estimated 324 species [67]. Eleven families have been included in coronavirus surveillance reports (Table 3). Several publications provided the total bats sampled within a study though may not have specified per species or country, and thus 1966 sampled bats could not be included [29,41]. The sample numbers (per species per country) were not specifically indicated in Anthony et al. [30], but total PREDICT surveillance data for the seven African-surveyed countries was accessed online from Healthmap.org and included in the analyses. We acknowledge that the data likely exceeds the sample size for the countries used for the analysis in the 2017 publication; however, we felt that including the data in our assessment greatly contributes to the total bats sampled in Africa per species—by over 10,000 individuals. Moreover, this data was also used in Table 2 and Tables S1–S3. Of the approximate 127 total bat species included in studies, bat coronaviruses were identified in 59. Nearly 26,000 bat individuals are estimated to have been tested for coronaviruses in African surveillance studies using one or more assays. However, this number comprises mainly pteropid and hipposiderid bats (41.8% and 33%, respectively) and varies per family. The table below highlights the need for additional surveillance in several families, such as the Vespertilionidae. These are abundant bats, and increasing the sample size tested of species in this family may provide a greater understanding of the host ecology of coronavirus species such as MERS-related viruses.

Coronavirus RNA has been detected in nine of the eleven families sampled, excluding the Emballonuridae and Rhinopomatidae. The Rhinopomatidae represents only one tested individual; approximately 678 bats from four species in the Emballonuridae family have been investigated (*Coleura afra, Taphozous perforates*, *Taphozous mauritianus,* and *Taphozous hildegardeae*). This includes surveillance from eight countries with sample sizes varying from 1 to 172 (Tables S4 and S5). Comparatively, coronaviruses have been identified from families like the Megadermatidae, Rhinonycteridae, or Nycteridae, from which far fewer individuals were analyzed (25–299). The lack of viral detection from the Emballonuridae family could be due to insufficient sample sizes, extremely low prevalence, time of sampling, highly diverse viruses missed by consensus primers, or the absence of coronaviruses. The remaining unsampled Myzopodidae and Cistugonidae families are small (two species each), with limited distributions in Madagascar and Southern Africa, respectively.

Primary surveillance reports investigating one or two species/genera typically focus on abundant hosts that may form large populations with frequent opportunities for contact with human communities [28,31,32,34]. Studies sampling many diverse genera/species (83% of primary surveillance reports) mostly sample species opportunistically present at one or more surveillance sites (Table S3). To estimate sample sizes per species, we looked at the total and average number of individuals per species tested in these reports and specifically noted sample sizes of less than ten individuals (Table S3). For some species, below ten individuals were tested, whereas several hundred [19,27,30,36,45,47] or even thousands of individuals from other species were sampled [44,46]. It was more common for less than 100 individuals to be sampled per species, though a few reports averaged 100–150 per species [19,27,30,36,45,47]. The percentage of species within a report for which less than ten individuals were sampled ranged between 18.5 to 100% of species (Table S3). This constituted more than 50% of species sampled from 11 of the reports and likely represented opportunistically caught individuals. This could not be determined for a further four reports, as sufficient detail was not specified, or samples collected represent colony or population-level sample collection.

A guideline for optimal sample sizes per species was proposed by the meta-analysis of coronavirus surveillance in 20 countries by Anthony et al. [30], with the optimal sampling number being approximately 397 individuals. This was calculated to detect the average number of unique coronavirus groups relating to probable viral species (2.67) estimated to be present in each bat species. Their findings identified that sampling less than 154 individuals per species constituted poor returns on investment and sampling effort [30]. The percentage of species per report from which coronavirus nucleic acids were detected varied between 8.3% to 66.7% (excluding when only one species was sampled). Overall, the percentage positivity of coronaviruses per total samples ranged from below 1% to 25.7% (excluding pools) (Table S3). As expected, increasing either sample sizes or number of species tested show correlation with increased positivity percentages (Pearson’s product correlation *t* = 8.9289, df = 21, *p <* 0.001 and *t* = 5.4952, df = 20, *p <* 0.001, respectively). The differences in positivity can be attributed to many factors, including the nucleic acid detection assay, the methodology for sample collection (preservation of nucleic acids), time of sampling coinciding with coronavirus excretion, species sampled, and sufficient sample numbers per species. Tables S4 and S5 highlight species commonly detected to host coronaviruses; a detailed description of ‘high-risk’ viruses identified from host species is described below.

#### 3.1.3. Importance of Accurate Bat Species Identification

Correct identification of bat species is essential to conclude potential virus-host associations and estimation of host-viral distribution ranges. This is especially important for complex bat species with similar morphological markers, such as members of the Hipposideridae, Rhinolopidae, and Vespertilionidae. Since the start of coronavirus nucleic acid surveillance among bat species in Africa in 2009, several bat species have undergone species reassignments and name changes. We could not update all new species names for this review and used the taxonomy described in the 2019 African Chiropteran report [67]. However, recent changes of note are among the Hipposideridae, Rhinolophidae, Miniopteridae, and Vespertilionidae families, with additions of new genera (*Afronycteris*, *Pseudoromicia, Vansonia* (elevated to genus)) and the reassignment of species to existing and new genera [68,69,70,71]. Some of these include *Hipposideros* species reassignments to the genus *Macronycteris* and the resolution of some *Neoromicia* species with reassignments to *Laephotis, Afronycteris,* and *Pseudoromicia* genera [68,69]. Currently recognized species may be accessed at www.batnames.org (accessed 18 November 2020) [72], and new species need to be correctly correlated to geographical distributions.

We investigated the methodologies for host identification implemented by the primary surveillance reports (Table S3). No identification methodologies for bat species were stipulated in seven (30%) of the bat coronavirus surveillance studies; five (22%) report the use of keys to determine morphological identities by either field teams, veterinarians, or experienced chiroptologists; and two (9%) report the use of molecular means of species confirmation. Only nine reports (39%) describe both morphological and molecular methods to identify and confirm host species (Table S3). Molecular methods include either mitochondrial cytochrome B gene or cytochrome C oxidase subunit I sequencing [73,74]. Not only is this good practice in ensuring accurate determination of host species identity, but if deposited on public reference databases, it ensures that the records of these sequences for sampled species are expanded. However, depositing sequences of individuals lacking accurate morphological identification and failure to update taxonomic changes generally leads to confusion and incorrect host reporting. Thus, reference material on these databases must be associated with correctly identified individuals where morphological identification was conducted by highly trained individuals or experienced bat taxonomists.

#### 3.1.4. Characterization of Bat Coronavirus Genomes and Virus Isolation Attempts

Bat coronavirus surveillance in Africa primarily focused on amplifying and sequencing short amplicon sequences and subsequent diversity determination. The majority of African bat coronaviruses are therefore unclassified and are only represented by a short-sequenced region. Further characterization of the detected coronaviruses is essential for improved phylogenetic placement and comparisons of various genes/proteins for phenotypic analyses. Studies aiming to further characterize identified coronaviruses employed diverse methodologies (Table S2). Sequence-specific primers have been successful in extending the sequenced regions of the ORF1ab [28,47] or recovering complete coding regions of structural genes like the nucleoprotein gene [27,37]. Sequencing these regions generally involved primer-walking strategies with conventional Sanger sequencing or even high throughput sequencing platforms to overcome the length limit of conventional sequencing. The informal RdRp gene grouping units (referred to as RGU; Figure 4) developed by Drexler et al. [66] amplifies an 816 nucleotide amplicon of the RdRp gene. The pairwise distances of the translated 816 nucleotide fragments (272 amino acids) have been used to delimit different groups as a surrogate system for taxonomic placement of detected bat coronaviruses that lack complete genomes. Grouping units of alphacoronaviruses differ by 4.8% and betacoronaviruses by 5.1% [61]. These grouping units have been used as an extension assay by 22% of African bat coronavirus studies [32,35,43,44,46]. It is worth noting that these units are an unofficial estimate of possible species groupings and may be subject to revision as new diversity is detected (as evident by previous decreasing betacoronavirus thresholds from 6.3% to 5.1%) [61].

The number of bat coronaviruses that can correctly be assigned to a viral species is limited to those with available complete genomes. From African studies, there are over 1840 partial coronavirus gene sequences available among public domains (such as NCBI’s GenBank), though only 13 complete genomes and 12 near-complete genomes [19,32,34,41,46,57,58,59]. The MERS-related *Pipistrellus* bat coronavirus from Uganda was recovered with unbiased sequence-independent high throughput sequencing on the MiSeq platform [59] and a near-complete genome of Zaria bat coronavirus from Nigeria using 454 pyrosequencing [41]. Sanger sequencing with classic primer-walking spanning the entire genome with 70 overlapping hemi-nested PCR assays was implemented to recover a MERS-related *Neoromicia* bat coronavirus from South Africa [58], with a second variant from the same host sequenced using 11 overlapping hemi-nested PCR assays on the MiSeq platform [32]. For more novel viruses, amplification of more conserved coronavirus genome segments with nested consensus degenerate primers are frequently required before being able to sequence more diverse regions with long-range PCRs [19,46,57].

The limited number of complete African bat coronavirus genomes are reflective of the challenges involved. These include the limited scope of certain studies, low viral RNA concentrations, unavailability of sufficient material, lacking related reference genomes for primer design, availability of high throughput sequencing platforms, expertise, and cost [32,37,46]. To overcome some of these constraints, such as limited availability of material, virus culturing can be attempted. However, coronaviruses are notoriously difficult to isolate in vitro, with various methodologies utilized (reviewed in Geldenhuys et al. [75]). Only bat coronaviruses closely related to SARS-CoV have thus far been successfully isolated in Vero cells because the bat viruses could use the same receptors as SARS-CoV [76,77]. This challenge and limited sample material available after nucleic acid extraction and high-biocontainment requirements are likely contributing factors to why none of the 23 primary surveillance publications or secondary characterization reports attempted cultivation of coronaviruses in cell culture (nor described attempts).

It is important to note the formats of naming conventions among bat coronavirus studies, with only some providing sufficient information on the origins of sequences (Table S2). The Coronavirus Study Group of the ICTV recommends adopting a standardized format for nomenclature that has been used for Influenza viruses and avian coronaviruses [6]. Namely, the reference to a host organism from which the viral nucleic acid was derived, the place of detection, a unique strain identifier as well as mention of the time of sampling (e.g., virus/host/location/isolate/date or as an example BtCoV/Neoromicia/RSA/UP5038/2015). This format also allows rapid identification of inter-genus viral sharing in phylogenetic trees and highlights similar clades of viruses occurring in related species independent of geography. More importantly, this naming convention makes no inference of belonging to a particular species, as species assignments may only be performed once the requirements have been met (i.e., sequencing the genome according to species demarcations).

#### 3.1.5. Coronavirus RNA Identified in African Bats

Global coronavirus surveillance in bats has established several generalizations, with which African studies are in agreement. Namely, bat coronaviruses generally display host specificity, which is usually evident at the genus-level [19,61,78,79,80]. As a result, certain viral species or even subgenera may be predominantly associated with specific host genera (e.g., rhinolophid bats and *Sarbecovirus*). This association has been observed to be independent of the geographical isolation of the bat hosts [38,81,82]. The evolution of coronaviruses has been suggested to involve a combination of two mechanisms, co-evolution between viral and host taxa and frequent cross-species transmission events [78]. Co-evolution is evident by genus-specificity and the large diversity of bat coronaviruses globally sampled, though many taxa host more than one species/group of coronaviruses [37,78]. Meta-analyses of publicly available bat coronavirus sequences confirmed long-term evolution among bats and determined that frequent cross-species transmissions occur, particularly among sympatric species, though often result in transient spillover among distantly related host taxa [19,30,78]. Such transmissions potentially create viral adaptation opportunities to new hosts and increase overall genetic diversity [83]. Uniquely for Africa, the genetic information of bat coronaviruses sharing similarity to human coronaviruses have been identified in four of the five subgenera associated with human coronaviruses—*Duvinacovirus, Setracovirus, Merbecovirus,* and *Sarbecovirus* (Figure 1A,B). Such findings suggest opportunities for transmission from bats to other animals or directly to humans may have occurred in the past. Though these viruses are still circulating among these hosts, discerning current risks of spillover is limited by available evidence.

Together with highly variable mutation rates [84,85], coronaviruses are also known for recombination events, where homologous recombination between similar coronaviruses is the most likely. However, recombination between different co-infecting coronaviruses from different subgenera/genera has also been documented [86,87,88]. Opportunities also increase when bats are co-infected by more than one species of coronavirus. Moreover, heterologous recombination between viral families has also led to the assimilation of novel genes in certain coronaviruses [86,87]. Recombination hotspots within the spike gene have been identified for diverse coronaviruses originating from humans, domestic animals, and bats [89]. Some of the new resultant variants may have improved fitness advantages within their native or new hosts, and new recombinants may be more suited to the usage of new receptor molecules.

Phylogenies were constructed with the sequences from the 23 primary surveillance reports and secondary characterization research studies, representing the sequence diversity of African bat coronaviruses compared to formally classified species and relevant reference sequences (see Appendix A and complete phylogenies in Figures S1 and S2). The following sections summarize the information available regarding detected bat coronaviruses associated with known human coronaviruses and highlight the importance of recombination in the emergence of novel viruses. We also discuss the large diversity of unclassified and unstudied viruses in some highly abundant host species and consider possible interaction opportunities between humans and bat hosts.

##### Alphacoronaviruses—*Duvinacovirus*, *Setracovirus*, and Unclassified Virus Relatives of Human Alphacoronaviruses

**Figure 5:**
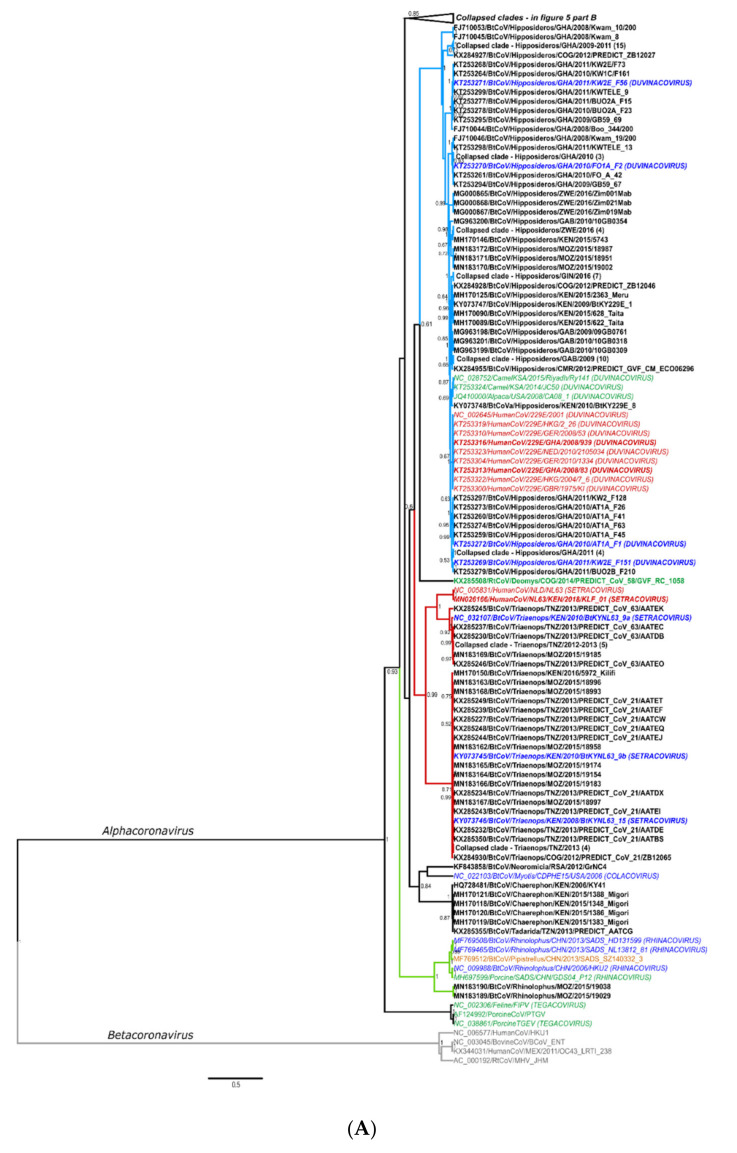

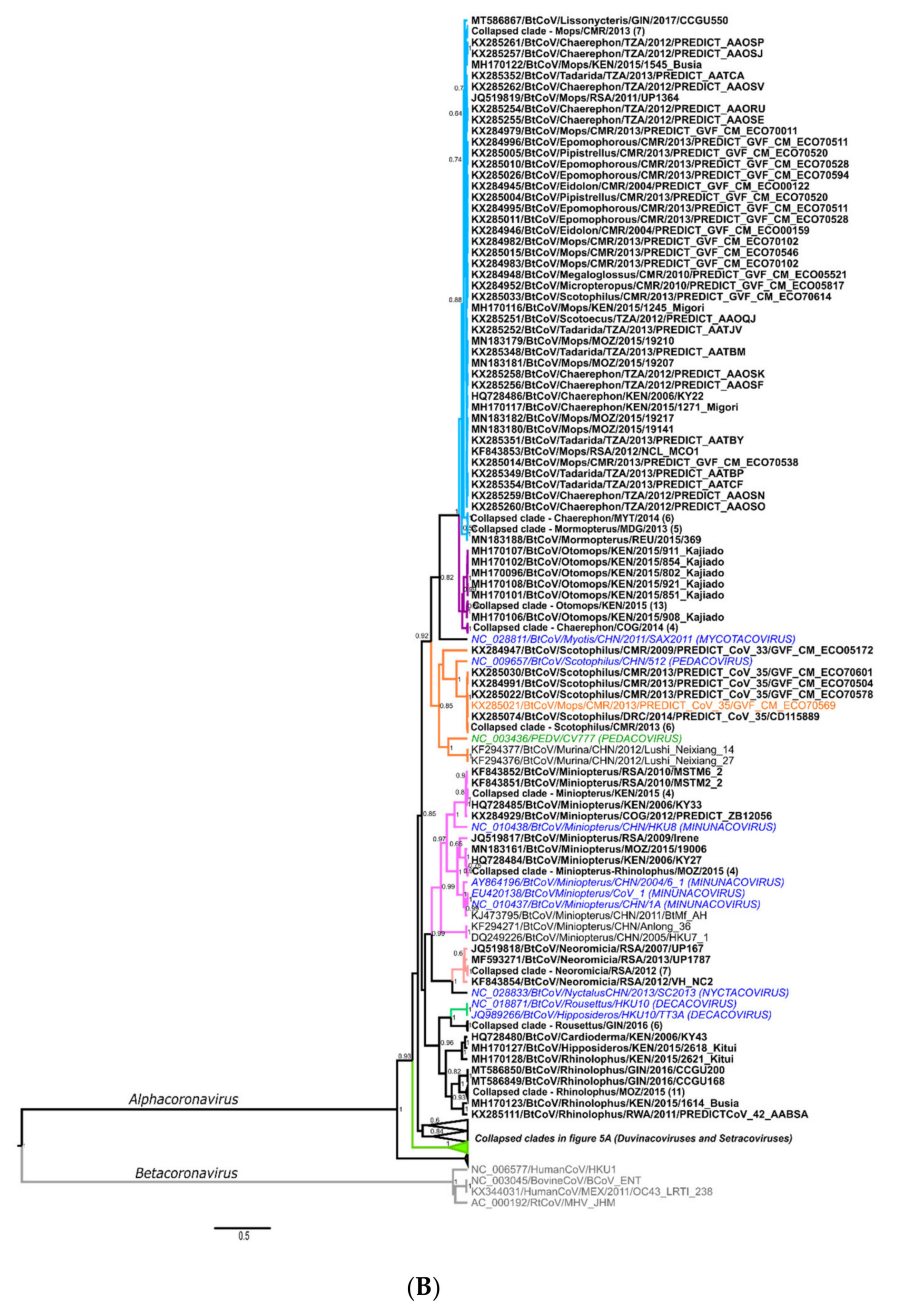
(**A**,**B**): Alphacoronavirus Bayesian phylogeny of the RdRp partial region (corresponding to approximately 15,200–15,400 nt of the coronavirus genome). Clades collapsed in A are shown in B (and vice versa). To include the maximum number of sequences, sequence lengths were trimmed to a generally useable length of 260 nucleotides. Sequences resulting in shorter lengths were omitted. Sequences in italics indicate formally recognized species (subgenera indicated in capital letters at the end of sequence names); sequences in bold originate in Africa; red highlights human viruses; green indicate non-bat animal hosts; blue/italics indicate formally recognized bat species; orange indicate viral detections from hosts not typically associated with a particular group of coronaviruses. All sequence names were edited to conform to the correct convention, with the modification of the unique sequence identifier listed last due to convenience. Only posterior probabilities of greater than 0.5 are indicated. No unpublished sequences are shown.

Several African bat coronaviruses share genetic similarity with the two human alphacoronaviruses, HCoV229E (*Duvinacovirus*) and HCoVNL63 (*Setracovirus*). As seen in Figure 5A, hipposiderid bats (genus *Hipposideros*) are associated with coronavirus sequences similar to HCoV229E and have been reported across a wide geographical distribution (Ghana, Kenya, Cameroon, Zimbabwe, Republic of the Congo, Rwanda, Uganda, Gabon, Mozambique, and Guinea) [19,29,30,31,37,38,39,45,46]. Due to taxonomic revisions and reassignments [69], the *Macronycteris* genus (Hipposideridae) may also be associated with duvinacoviruses (Table S5).

Full genomes of four *Hipposideros* alphacoronaviruses from Ghana were compared to current and historical isolates of HCoV229E and an alpaca coronavirus (similar to HCoV229E) from the USA [46]. Sufficient similarity was found between genomes to consider them members of the same *Human coronavirus 229E* species within the *Duvinacovirus* subgenus. The analysis suggested multiple recombination events have occurred among genomes, including gene losses (e.g., ORF8 within human viruses) and deletions within the spike gene [46]. Several of the bat viruses with similarity to HCoV229E for which no complete genomes are available indicate that there are sequence divergences of approximately 13.5% among RdRp partial gene segments, suggesting circulation of highly diverse HCoV229E-related viruses. The scenario would suggest that HCoV229E may have originated from the large diversity of *Hipposideros* HCoV229E-related bat coronaviruses in the past 200 years (based on the current sequence diversity), with camelids (alpacas, camels, etc.) as possible intermediate hosts [46].

Similarly, several African bat sequences cluster around HCoVNL63 (Figure 5A) and originate from the genus *Triaenops* (Rhinonycteridae family). *Triaenops afer* is the only mainland Africa species currently recognized within the genus after it was split from *T. persicus*, which only occurs in the Middle East [67,90] (with *Triaenops menamena* from Madagascar). Partial and complete genomes were first reported in Kenya [19] with additional partial genomes from the Republic of the Congo, Tanzania, Mozambique, and Madagascar [30,38] (Table S4). Three full genomes were recovered from Kenyan *T. afer* bats and compared to HCoVNL63 [19]. Much like 229E-related bat viruses and HCoV229E, comparisons of the bat viruses to HCoVNL63 identified additional ORFs (ORFx) in bat viral genomes that were absent in HCoVNL63 [19]. The new species, *NL63-related bat coronavirus strain BtKYNL63-9b* (*Setracovirus*), comprised of *Triaenops* coronavirus strains, has been recognized. *Triaenops* virus 9a shares the closest similarity to HCoVNL63 with 78% overall nucleotide identity. The spike was the most divergent gene, with gene phylogenies showing the spike gene of HCoVNL63 grouping with *Hipposideros* 229E-related bat viruses detected in the same study [19]. Recombination analysis of HCoVNL63 indicates multiple breakpoints within the spike gene and suggests a history of recombination between the *Triaenops* NL63-related viruses and *Hipposideros* 229E-related viruses giving rise to the lineage of HCoVNL63 before its introduction into human populations [19]. As with HCoV229E, an intermediate host (and not bats directly) may likely have been involved in introducing progenitor HCoVNL63 viruses into the human populations. Such intermediate hosts are often domesticated livestock animals (such as camelids in the case of HCoV229E) as they have more frequent contact with people, underscoring the need for expansive surveillance within domestic animals to complement surveillance in wildlife.

Bats from the *Hipposideros, Myonycteris* and *Triaenops* genera are all small insectivorous bats and have many overlapping ecological features in terms of habitat. *Hipposideros* and *Myonycteris* primarily roost in caves, though certain species have been known to roost in rock crevices, under bridges, and in tunnels [67]. *Triaenops* have been found roosting in small trees and certain shrubs and mines and caves [91]. Moreover, bats from all three genera are sensitive to human activities that lead to habitat loss and roost disturbance [67]. The surveillance findings show that these viruses continue to circulate in these hosts, with the potential to recombine and create new variants. Establishing whether these viruses pose possible zoonotic risks is limited due to lacking evidence. In vitro studies can assist with determining permissivity or pathogenicity in different cell lines, and protein modeling can suggest the likelihood of receptor binding of bat viruses in spillover hosts. There is also a lack of nucleic or serological investigations into potential spillover animal species that overlap with the bat hosts’ geographical distributions and ecological niches.

##### Alphacoronaviruses—Molossids and a Large Diversity of Uncharacterized Bat Coronaviruses

The diversity of bat alphacoronaviruses from Africa is high. Much of the reported sequences share genetic similarity to members of described subgenera, such as *Rhinacovirus*, *Pedacovirus*, and *Minunacovirus* (Figure 5B). Many of the other sequences represent undescribed diversity and may possibly belong to new subgenera. A large number of unclassified alphacoronaviruses have been identified from molossid bats (Figure 5B). Generally, these sequences form three clades, with sequences similar to a species of *Colacovirus* detected in *Chaerephon* and *Tadarida*; a sister clade of the *Mycotacovirus* subgenus that split into an *Otomops*-specific species clade from Kenya; a predominantly *Mops*/*Chaerephon* group of alphacoronaviruses from several countries (Cameroon, Kenya, Tanzania, South Africa and the Republic of the Congo). The latter group also contains a large volume of sequences from various pteropid species (as well as a few vesper species) from Cameroon [30], making it a mixed family clade or a group of viruses frequently prone to host switching. Sequence information on these viruses largely constitutes short sequences from surveillance assays as well as a few partial genomes (HQ728486/BtCoV/*Chaerephon*/KEN/2006/KY22 and HQ728481/BtCoV/*Chaerephon*/KEN/2006/KY41) [57]. These coronaviruses were detected from molossid species such as *Chaerephon pumilus, Mops condylurus, Otomops martiensseni,* and *Tadarida aegyptiaca,* with only 16 of the 44 species from the Molossidae family having been included in surveillance studies. Of note are recent taxonomy changes among this family [72]. Moreover, as indicated in Table S4, large numbers of molossid bats tested are only specified to genus level, with nearly 171 *Chaerephon* spp., 30 *Mops* spp., and 64 *Tadarida* spp. reported. This again reiterates the need to identify hosts down to species level. These species are highly abundant with widespread distributions throughout Africa and are often encountered in urban settings. They are frequently found to be roosting in large populations (several hundred) in the rafters or roofs of buildings such as houses or public institutions like schools, universities, and libraries [67]. As a result, opportunities for contact arise between bat excreta and people (and domestic animals). Though there is no current zoonotic association with these coronaviruses, their abundance among a commonly encountered bat species, with possibly frequent exposure opportunities warrant investigation. Significant characterization of these viral groups is required to better understand this diversity and investigate the zoonotic potential of these alphacoronaviruses.

##### Betacoronaviruses—Merbecoviruses and Vespertilionid Bats

MERS-CoV emerged on the Arabian Peninsula in 2012 and is now considered endemic to the region due to the presence of the primary reservoir, the dromedary camel [7,92,93]. According to reports from Africa, Europe, Asia, and even South America, viruses sharing similarities to MERS-CoV (*Merbecovirus*) are associated with more than one bat host genus or family [32,43,44,59,83,94,95]. The MERS-related coronaviruses genomes currently sharing the highest similarity to human and camel MERS-CoV were detected in Africa from *Neoromicia capensis* (South Africa) and *Pipistrellus hesperidus* (Uganda) [32,58,59]. Both *Neoromicia* and *Pipistrellus* are small insectivorous bats belonging to the Vespertilionidae family, with several species reassignments occurring in 2020 [68]. Due to taxonomic rearrangements, the genera *Laephotis, Afronycteris,* and *Pseudoromicia*, necessitate inclusion into future MERS-related coronavirus surveillance due to possible intra-host sharing of coronaviruses. Sampling efforts into the previously recognized *Neoromicia* species include approximately 238 individuals and only 100 individuals among *Pipistrellus* species (Table S4), warranting intensified surveillance. According to published reports, very few individuals have been found to harbor MERS-related viruses from these bats sampled.

The three available viral full genomes recovered from *Neoromicia (Laephotis)* and *Pipistrellus* were used to classify the viruses as belonging to the same viral species as human and camel MERS-CoV. Within the bat-borne MERS-related viral genomes, the spike genes shared the lowest similarity to human and camel MERS-CoV spike genes (approximately 63–64% nucleotide identity) [32,58,59]. The latter viruses utilize the DPP4 (Dipeptidely peptidase 4) as an entry receptor. Using homology models based on the crystalized structure of the spike protein of the *Pipistrellus* MERS-related virus, Anthony et al. [59] determined that the bat virus spike was unlikely to utilize DPP4 due to insufficient similarities among the required residues to facilitate binding of the spike to the receptor. This was practically demonstrated when recombinant MERS-CoV particles containing the spike from the *Pipistrellus* MERS-related virus were unable to enter Vero cells (unlike wild-type MERS-CoV) [59]. Moreover, recombination analysis also identified potential breakpoints within the spike gene for *Neoromicia (Laephotis)* MERS-related virus PML/PHE1 and *Pipistrellus* MERS-related virus PREDICT/PDF-2180 [58,59]. The data thus suggests that the identified bat-borne MERS-related viruses have not served as direct progenitors of MERS-CoV detectable in camels and humans, though whether recombination occurred in a bat host or an intermediate host is uncertain.

Depending on the species, both *N. capensis (reassigned as Laephotis capensis)* and *P. hesperidus* have widespread distributions in various parts of Africa [67,68]. *N. capensis* (*L. capensis*) is an abundant and adaptable species distributed from sub-Saharan Africa to South Africa. They typically roost under bark or rock crevices that limit roost sizes to a few individuals [96]. However, these bats have adapted to occupy increasingly available urban roost sites such as cracks in walls and the roofs of houses, which allow populations over 50 individuals to congregate [96,97]. As a result, *N. capensis (L. capensis)* is a common species in urban areas that beneficially aid in decreasing insect populations attracted by city lights. Conversely, *P. hesperidus* is not very abundant and sparsely populated within its distribution from sub-Saharan Africa (Ethiopia down) to South Africa [98].

##### Betacoronaviruses—Sarbecoviruses with African Rhinolophids

**Figure 6:**
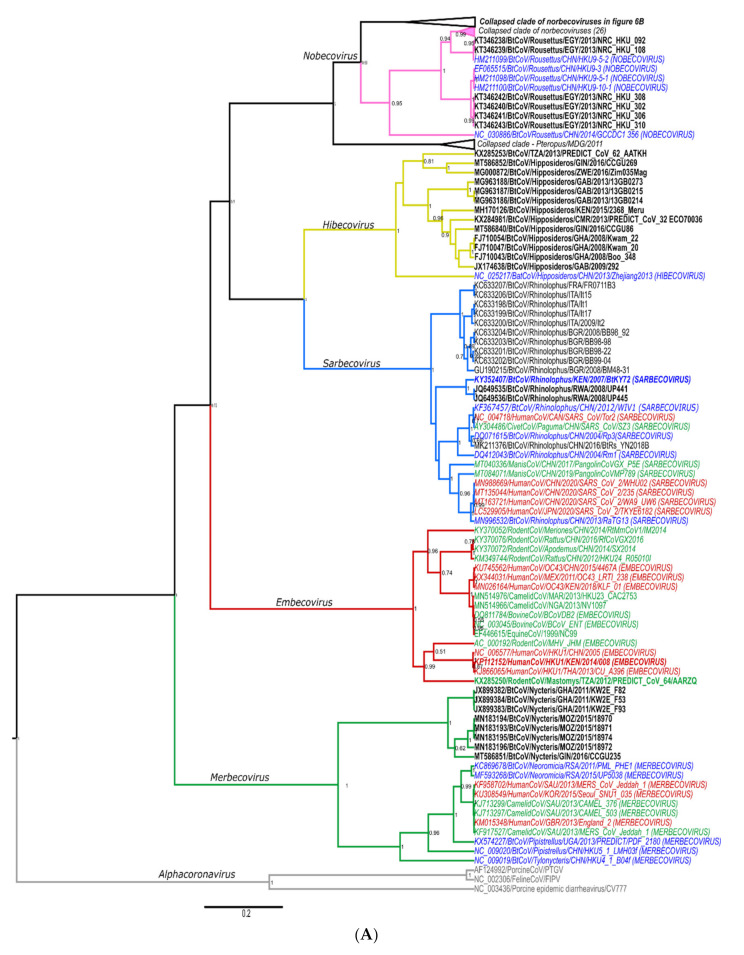

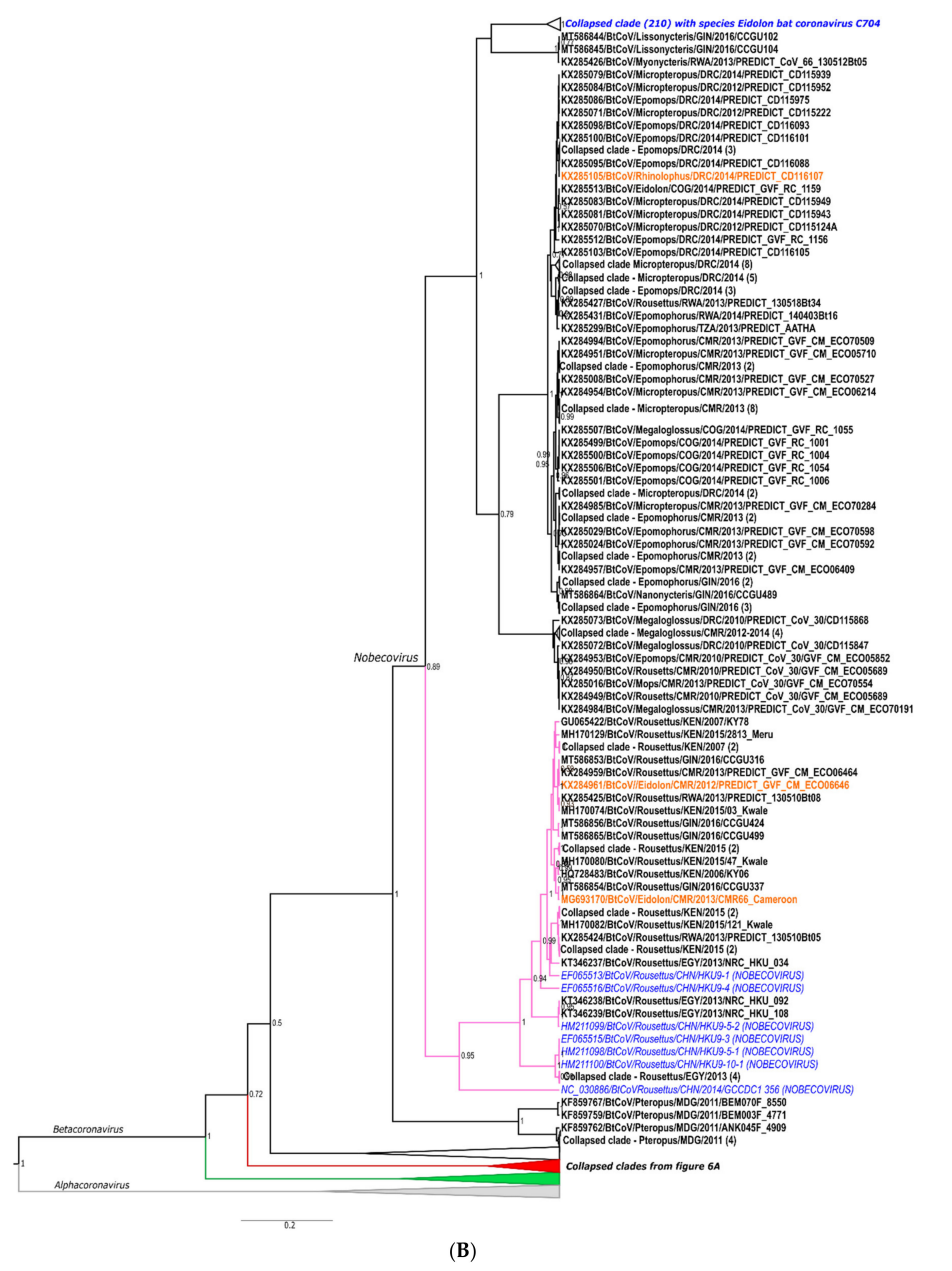
(**A**,**B**): Bayesian *Betacoronavirus* phylogeny of a 294-nucleotide sequence region of the RdRp gene. Shorter sequences were omitted. Clades collapsed in A are shown in B (and vice versa), and the collapsed clade of *Eidolon* nobecoviruses may be viewed in Figure S2). Sequences in italics indicate formally recognized species (subgenera are indicated in capital letters at the end of sequence names); sequences in bold originate in Africa; red highlights human viruses; green indicate non-bat animal hosts; blue/italics indicate formally recognized bat species; orange indicate viral detections from hosts not typically associated with a particular group of coronaviruses. All sequence names were edited to conform to the correct convention, with the modification of the unique sequence identifier listed last due to convenience. Only posterior probabilities of greater than 0.5 are indicated. No unpublished sequences were included.

Bat coronavirus sequences sharing similarity to human sarbecoviruses (SARS-CoV and SARS-CoV 2) have been identified throughout the geographic distribution of rhinolophid bats in Asia, Europe, and Africa. The highest genetic similarities between human and bat sarbecoviruses (Rp3, HKU3, WIV1, WIV16, ZXC21, ZC45, RaTG13, RmYN02) originate in Asia [5,76,77,82,99]. Bat species from the *Rhinolophus* genus are considered the main hosts for the genetic diversity of bat sarbecoviruses [16,66,88]. Some species occurring in Europe have also been reported from Northern Africa, such as *Rh. ferrumequinum* and *Rh. euryale*; and are known hosts of sarbecoviruses [66], but very few sequences with similarity to members of the *Sarbecovirus* subgenus have been identified in Africa (Figure 6A). Reports include partial RdRp sequences from two species (*Rh. hildebrandtii* and *Rh. clivosus*) from Kenya, Rwanda, and Uganda (non-universal surveillance region) with similarity to SARS-CoV [19,35,36,100]. Further sequencing of the complete genome of BtCoVKY72 detected from a *Rhinolophus* sp. from Kenya identified the virus as a member of the *Severe acute respiratory syndrome-related coronavirus* species within the *Sarbecovirus* subgenus [100].

This limited detection of sequences similar to sarbecoviruses may be due to lacking surveillance of individuals within the *Rhinolophus* host genus. There are 38 extant *Rhinolophus* species in Africa, with approximately 728 individuals from 14 species included in published surveillance efforts from 11 countries (Table S4). However, very small sample sizes averaging between 1–62 individuals have been tested per species. To our knowledge, no bat coronaviruses sharing high similarity to the SARS-CoV 2 clade sarbecoviruses have been identified from African bats. In addition to betacoronaviruses, unclassified alphacoronaviruses have also been identified from four *Rhinolophus* species, suggesting large diversities of coronaviruses to be present in these bats [19,29,33].

Rhinolophids are taxonomically challenging to identify with frequent revisions to species due to highly convergent morphology [67]. Certain species are widespread and have distributions spanning into other continents, such as *Rh. clivosus* from Africa and into South West Asia [101]. These bats generally roost in caves, unused mines, and buildings [67] and are threatened by disturbances to roosts such as mining and the use of pesticides and insecticides [102], though provide valuable ecosystem services by decreasing the populations of crop-damaging insects [102].

Sequences with similarity to sarbecoviruses have also been reported from non-rhinolophid genera, including *Chaerephon* spp. in Kenya and hipposiderids in Rwanda, Cameroon, and the Republic of the Congo [26,30,36]. The latter hosts’ detections were few and may represent transient spillover between hosts (*Rhinolophus* and *Hipposideros*), possibly co-roosting. In addition, some other studies have reported the detection of viruses with homology to SARS-CoV in hipposiderid bats, though these viruses were part of a more distant sister clade than rhinolophid SARS-related viruses. Moreover, this sister-clade was later formally classified as the *Hibecovirus* subgenus. Due to the thorough surveillance of hipposiderid bats, these viruses have been reported from various countries, including Ghana, Gabon, Nigeria, Kenya, Rwanda, Zimbabwe, Guinea, and Rwanda (Tables S4 and S5).

##### Betacoronaviruses—Nobecoviruses and Fruit Bats

Members of the *Nobecovirus* subgenus are not currently associated with any known zoonotic diseases, though much like the aforementioned molossid alphacoronaviruses warrant further investigation due to their widespread occurrence in several abundant fruit bat species [79]. Nearly two-thirds of all the unclassified sequences in Figure S2 likely represent members of this subgenus. Described species in this genus include two Asian bat viruses, *Rousettus bat coronavirus HKU9* and *Rousettus bat coronavirus GCCDC1* detected in species such as *Rousettus leschenaultia* [80,103], as well as *Eidolon bat coronavirus C704* in Cameroon [34]. The African detections sharing similarities to members of the *Nobecovirus* subgenus are indicated in Table S5. These detections have been widespread and predominantly reported from fruit bat genera such as *Rousettus, Eidolon*, *Micropteropus, Epomophorus, Pteropus, Epomops, Myonycteris* (formerly *Lissonycteris*), and *Megaloglossus* [19,26,27,28,29,30,34,36,39,47]. Additionally, similar sequences have been reported from several insectivorous bat species, though whether these represent active maintenance of the virus in these hosts or transient spillover is unclear. Recombination events have been detected between species of the *Nobecovirus* subgenus identified in *R*. *leschenaulti* in Asia and rotaviruses (*Reoviridae;* double-stranded RNA viruses) co-infecting the same species, leading to the acquisition of the P10 orthoreovirus fusogenic gene [86].

*E. helvum* migrates over large distances throughout much of sub-Saharan Africa (Senegal to Ethiopia and down to southern Africa) and are tree-roosting fruit bats that form aggregates of thousands to millions of individuals. Large urban colonies have been recorded in trees of various cities (e.g., Accra in Ghana) [67]. Excreta from these urban colonies would provide ample opportunities for human contact with contaminated fecal and urine. *E. helvum* is also heavily harvested for bushmeat, with estimates of 128,000 bats being sold per year in markets in Ghana alone [67,104]. *R. aegyptiacus* also has a broad distribution throughout sub-Saharan and parts of Northern Africa, as well as South East Asia and the Western Palaearctic region [67]. This species is a cave-dwelling fruit bat that forms large colonies in the thousands (e.g., 5000 to 50,000), and may co-roosts with multiple insectivorous bat species. Opportunities for contact and possible viral sharing may thus arise between different bat genera, though possible exposure events to humans are more infrequent and generally arise due to human activities. These bats are often threatened by farmers who view fruit-eating bats as destructive to their crops as well as due to mining and other cave disturbances [67,105].

#### 3.1.6. Investigating Factors Affecting the Maintenance of Bat Coronaviruses

Understanding how bat coronaviruses are maintained in their host populations allows determination of infection duration and times that may be at ‘higher risk’ for coronavirus spillover opportunities. ‘High risk’ periods coincide with increased excretion of viruses from bats in a colony and may be associated with reproductive or seasonal factors affecting the viral infection dynamics of the colony. For example, an increase of mating activity and accompanying hormonal changes may affect the susceptibility of hosts to infection, or the increase in immunologically naive juveniles at the start of a birthing pulse creates a large population of bats susceptible to infection [106,107,108]. Understanding these dynamics allows the formulation of management plans to mitigate risks and facilitate engagement with communities at risk of frequent contact with particular bat populations. Behavioral changes may assist in reducing the associated risks of exposure and possible spillover interactions [109].

Limited African studies (only 5) expanded data analyses to include correlations between bat biology, ecology, and viral status of hosts [30,36,38,40,44]. Those investigating increased infection among age classes agree that subadults are more likely to host coronaviruses than adults [30,36,44], consistent with reports from other continents [109]. A higher frequency of infection was also identified among lactating females [44], though also males [30]. Most disagreements center around seasonality, with either no correlation identified [36] or a higher chance of detecting coronaviruses in the dry seasons [30]. Longitudinal surveillance projects would be able to assist with such interpretations in the future.

Bats occupy a wide range of niches, including diverse roost preference (e.g., cave-dwelling or tree-roosting), eating habits (frugivores, nectivores, insectivores, etc.), population sizes (less than 10 to thousands), and level of social interaction between the same and different species (gregarious or non-gregarious). It may also be possible that factors affecting the maintenance of coronavirus infection among bat species may not be universal to all bat species. Thus, combining coronavirus data from different species may result in biased conclusions. For example, it has been suggested that bat coronaviruses may amplify within maternity colonies [108], though the reproductive seasons of diverse bat species do not all overlap, and certain species are capable of reproducing more than once a year, depending on the geographic regions. For example, *Rousettus aegyptiacus* displays two birthing pulses among populations along the North of Africa [110], while populations in Southern Africa have only one [111]. Thus, if coronavirus maintenance is linked to its host species’ reproductive biology, viral shedding may be predictable for certain species in particular climate zones.

A recent study predicted high-risk periods for different host species utilizing available surveillance data from three countries (Rwanda, Uganda, and Tanzania) and Bayesian modeling [30,60]. Though several assumptions were made regarding the duration of lactation and weaning, they determined that juveniles recently weaned were 3.34 times more likely to shedding coronavirus RNA than juveniles that were not recently weaned. Even adults were nearly four times more likely to be shedding coronaviruses when juveniles were being weaned [60], possibly due to increased coronavirus excretion levels within the colony. As described in Wacharapluesadee et al., [109], increased coronavirus shedding among juvenile bats may be due to vertical transmission from mother to pup, which coincides with studies describing viral shedding from lactating females with increased frequency compared to non-lactating females [112]. The higher frequencies observed in recently weaned juveniles may be due to the loss of maternally received antibody protection following weaning [60,108]. These conclusions require confirmation with longitudinal surveillance among investigated bat species as well as serological studies determining changing antibody levels between lactating mothers, weaning and non-weaning juveniles, as well as other adults in the colony.

### 3.2. Surveillance in Other Wildlife and Domestic Animals (Livestock)

Coronavirus nucleic acid surveillance among non-bat wildlife, livestock, or other domestic animals in Africa is very limited, both in the frequency of research, sample sizes of animals tested, locations targeted, and are frequently investigated for only specific coronaviruses. Nucleic acid testing in animal populations where the prevalence of infection may be very low would yield limited data, provided that sampling was performed at a time when animals are infected or actively excreting viruses [113]. We only identified four reports in which other animals were tested for coronavirus nucleic acids, including anthroponoses of HCoVOC43 between humans and chimpanzees in Côte d’Ivoire [48], MERS-CoV specific surveillance among 4248 livestock animals from Ghana (cattle, sheep, donkeys, goats, and pigs) [50], general surveillance among 731 wildlife animals (rodents, non-human primates, and ad hoc samples of other wildlife) in Gabon [40], as well as just over 27,000 animals (birds, domestic animals, carnivores, pangolins, swine, rodents, and non-human primates) as part of the PREDICT surveillance initiative (accessed via Healthmap.org) (Table 4). Though this seems like a significant number of individuals tested, the total species diversity among all 16 countries sampled is much larger than the fraction represented by this surveillance. Moreover, not all hosts listed were surveyed in all countries (Table 4), with mostly opportunistic sampling from accessible individuals. However, even though the total positives detected in relation to the total number sampled is <1%, it still shows the presence of coronaviral RNA from among non-human primates (14 chimpanzees), ungulates (1 bush duiker), carnivores (1 African palm civet) and rodent species (13 individuals) from opportunistic surveillance [48,56].

Two of these sequences, publicly available and corresponding to the universal surveillance region (excluding the anthroponoses of HCoVOC43 from the chimpanzees), were included in the phylogenies in Figure 5A and Figure 6A (KX285508 and KX285250). Most of the detected African rodent coronavirus partial sequences are phylogenetically placed in the *Embecovirus* subgenus, with human coronaviruses OC43 and HKU1 and other rodent coronaviruses from Asia [18,30]. Divergent rodent alphacoronavirus virus RNA was also identified (KX285508), as well as highly divergent shrew coronaviruses [30]. The sequence information confirms surveillance data from Asia and Europe, namely that rodents and shrews likely harbour additional undiscovered diversity of coronaviruses. Improved systematic and longitudinal surveillance of wildlife and domestic populations will provide more data on the presence of coronaviruses among these animal groups. The research is too limited to make any conclusions regarding the absence of viral sharing between animal groups. Additionally, serological surveillance would complement nucleic acid surveillance by providing data on hosts not actively infected with coronaviruses.

Not included in Table 4 is the expansive surveillance of dromedary camel populations for MERS-CoV. MERS-CoV is not only endemic to the dromedary camel populations of the Middle East but also populations in Northern Africa (Burkina Faso, Ethiopia, Kenya, Mali, Morocco, Nigeria, Somalia, Sudan, Tunisia) [93,114]. Seroprevalence of adult dromedaries is high (80–100%) and may result in respiratory disease with viral shedding via nasal discharge [93,115]. Despite this widespread occurrence, MERS infections among people from camels have only been reported from the Arabian Peninsula [114,115]. Viruses from African dromedaries form a separate basal lineage to the two clades of MERS-CoV identified from infected people and camels on the Arabian Peninsula [114,116], though still share antigenic similarities through cross-neutralization [114]. Furthermore, within this African clade, genomes from the West and North African dromedary populations (Nigeria, Burkina Faso, Morocco) display deletions in specific accessory genes [114,117,118]. It has been suggested that these accessory genes are not required for the adaptation of the virus to dromedary camels and may have been necessary for a more historical host [114]. Whether bat-borne MERS-related viruses established in dromedary camel populations can only be addressed with better surveillance of African bat and dromedary populations, especially where bat and camelid distributions overlap [32].

## 4. Coronavirus Serosurveillance

Coronavirus serology is complex and faces several challenges—even among human coronaviruses [113]. Serological targets include the immunogenic nucleoprotein that is abundant during infections and the spike protein that allows for the detection of more specific antibody responses and neutralizing antibodies [119]. Targeting a more conserved protein (such as the nucleoprotein) may yield high seropositivity levels due to potential cross-reactivity of conserved epitopes among related coronaviruses, without being able to discern between different viral species (or genera). Depending on the assay target, cross-reactivity could complicate human coronavirus assays due to conserved motifs between seasonal human coronaviruses, SARS-CoV, and MERS-CoV [113], as well as between SARS-CoV and SARS-CoV 2 [120]. Serosurveillance among animal populations is similarly hampered with cross-reactivity as they may be exposed to unidentified coronaviruses. Due to the challenges of cultivating certain animal coronaviruses, virus neutralization tests to exclude cross-reactions are not readily feasible. A lack of specific animal coronavirus assays often leads to the use of human coronavirus assays (generally based on the spike protein). However, interpreting the results should be made with caution as cross-reactivity to unknown epitopes and modifications to validated assays may allow for false assumptions [113]. There is a great need to develop suitable assays for serological surveillance of diverse coronaviruses in wildlife and domestic animals. The lack of well-characterized reference sera to determine cut-off thresholds and limited species-specific biologics also challenges new assay development.

Bat coronavirus serology is demanding for all the aforementioned reasons and is further complicated by the large diversity of bat coronaviruses. Of note is that not all bat coronaviruses utilize the same receptor molecules. Angiotensin-converting enzyme two or ACE2 is the known receptor for SARS-CoV, SARS-CoV 2, and only the most closely related bat sarbecoviruses. The receptor-binding regions and important motifs even differ greatly between SARS-CoV and SARS-CoV 2 (see Andersen et al. [13]). The spike receptors for the larger majority of bat sarbecoviruses lack the required binding sites and are largely incompatible with human ACE2. The spike proteins of BtCoVKY72 only share 68–72% amino acid similarity to the spike proteins of SARS-CoV and SARS-CoV 2 and their most closely related bat viruses (unpublished data). Though, protein similarity alone cannot be used to determine if cross-reaction will occur due to the glycosylation and conformational folding of spike proteins [113].

In comparison to the number of studies investigating bat coronavirus nucleic acid surveillance, minimal serosurveillance studies have been performed on the continent. These include mainly Muller et al. [121], wherein a SARS-CoV ELISA kit with minor modifications was used to tests bat sera from the Democratic Republic of the Congo (DRC) and South Africa, as well as a MERS-CoV pseudo-particle neutralization assay to test *Rousettus* sera in Egypt and Lebanon by Shehata et al. [27]. Though no MERS-antibodies were detected in *Rousettus aegyptiacus*, antibodies reactive to SARS-CoV antigens were identified in 6.7% of bats tested (7 of 26 species) from the DRC and South Africa. These species include pteropid bats (*Rousettus, Myonycteris,* and *Hypsignathus*) as well as other insectivorous bat genera like *Mops, Miniopterus,* and *Rhinolophus*; many of these genera have since been identified to host either alpha- or betacoronaviruses. The results were confirmed with western blots, though no neutralizing antibodies were identified [121], cross-reactivity between potentially related bat coronaviruses. Increased bat coronavirus serological surveillance would provide better overall estimates of population exposure levels [119,122] and reduce false-negative assumptions from non-actively shedding hosts.

Wildlife, livestock or domestic animal serological surveillance in Africa is more frequent than serological surveillance among bats. A broad search of the literature found mainly studies focused on MERS-CoV serology and dromedary camel populations among various countries (reviewed in Dighe et al., [93]). Among domestic animals, several studies investigated livestock in Ghana [49,50,51]. Bovine coronavirus was determined to possibly be widespread among ruminants such as cattle and capable of spilling over into sheep and goats [51]. Cattle, sheep, goats, donkeys, and swineherds have been found lacking any serological response toward merbecoviruses like MERS-CoV or the similar *Nycteris* bat betacoronaviruses [50], or indeed HCoVNL63 and related bat viruses [49]. The authors highlight the need for such surveillance to be conducted in countries such as Kenya, where similar viruses to HCoV229E or HCoVNL63 were identified in bats.

Limited serosurveillance has been performed in wildlife. Though no feline coronavirus serological responses were identified among 13 lions from Botswana sampled between 2012 and 2014 [123], feline coronaviruses (particularly the highly pathogenic feline infectious peritonitis virus) have historically been shown to be actively circulating among captive cheetahs in the USA and free-living cheetah populations from Eastern and Southern Africa [124,125]. This lack of thorough surveillance in animals that may act as intermediate hosts and detecting spillover infections creates a gap in data not only for Africa but globally. Moreover, to our knowledge, no studies have investigated human populations in Africa for serological responses to bat coronavirus spillover [126].

## 5. Factors Associated with the Potential Emergence of Coronaviruses

Opportunities for potential pathogen exposure between humans and animals, including wildlife, are increasing. In Africa, the main factors include deforestation, agricultural intensification, and the collection, hunting, and butchering of bushmeat [22,127,128]. Interactions that are more specific to bats include ecotourism, mining, guano collection for fertilizer [128], or bat species that roost in man-made structures, such as houses, warehouses, schools, etc. Coronavirus nucleic acids are still detectable in guano fertilizer several days after collection, even if kept at room temperature (though viral isolation was not attempted) [129]. Although some factors may create opportunities for spillover, the exact routes of transmission are not yet clear. [130]. Research investigating potential interfaces in Africa is limited.

The bushmeat trade represents one of the most prominent points of contact between humans and bats on various continents [131], though it may practically represent a low risk of transmission for coronaviruses. Bushmeat serves as an important source of protein and household income in many African, Asian, and South American countries [132,133]. Large bats from the *Eidolon* or *Hypsignathus* genera are predominantly hunted, though smaller bats (*Hipposideros, Rhinolophus,* and *Myotis,* among others) are not excluded [133]. For sub-Saharan Africa alone, 52 African bat species (Table S4) are reportedly hunted in countries across their distribution [133]. *Alpha-* and *Betacoronavirus* sequences have been reported from at least 12 and 14 of these bat species, respectively (Table S4). Notably, viral sequences putatively grouping within the *Duvinacovirus*, *Sarbecovirus,* and *Hibecovirus* subgenera have been detected in one of the hunted bat species, namely *Hipposideros ruber* [30,36,37]. A large number of species deemed as bushmeat have, however, not been included in any coronavirus surveillance studies, and thus, their propensity as viral hosts and associated risk to humans remains to be determined. 

Live animal markets have been labelled as an ideal interface for human exposure and disease emergence and have been scrutinized due to the ongoing global COVID19 pandemic [134]. As in specific regions of Asia where such markets are commonplace, live or cooked bats are sold in selected African countries [104,135,136]. These bats may also be used in traditional medicine. Additionally, festivals in Africa focused on bats, such as those in Buoyem (Ghana) and Idanre (Nigeria), may provide opportunities for viral spillover [137,138]. The emergence of SARS-CoV and the SARS-CoV 2 pandemic has led to the banning of wet markets from selling live animals in China [132]. Both bans were eventually lifted and remains a point of debate [139,140].

Human-bat interactions are motivated by social, economic and cultural drivers, which form an integral part of infectious disease research. Different cultures have multifaceted perspectives concerning bats, which may be shaped by the local beliefs, use in traditional medicine, knowledge of bat biology, disease risk, or change during periods of food shortages [141,142,143,144]. Though limited information is available in Africa, several recent studies have considered the risk perceptions of human populations to bats and their associations with zoonotic diseases [142,143,144,145,146,147]. Overall, the results suggest that communities have limited knowledge of bats and do not generally perceive bats as a threat [142,143,145]. These perceptions may likely have changed following the COVID-19 pandemic.

With the known diversity of coronaviruses in bat species from Africa and the association of a number of these bats in human activities, exposure to these viruses is inevitable. There have to date not been any reports of novel coronavirus-associated diseases speculated to be of bat origin on the African continent, contrary to the link between bats and sarbecoviruses from Asia [5,16]. There is a clear overlap between practices in Asian and African countries with regards to animal trade. An intricate relationship between the factors associated with disease spillover from bats to humans is likely involved. Identifying the synergistic effects of these factors is simply the first step in understanding their roles in disease emergence.

## 6. The Future of Coronavirus Surveillance

The majority of African coronavirus surveillance has been focused on nucleic acid detection, estimating the genetic diversity of coronaviruses from bats and largely excluding other wildlife. Very limited epidemiological information is available to understand and support current assumptions regarding coronavirus maintenance among bat populations (effects of reproductive biology and ecologic impacts). Surveillance among other wildlife species and domesticated animals is so limited that no further conclusions can be reached on their risks. It is clear that bats host the genetic diversity of coronaviruses [17,18,78], but surveillance should be expanded to other species that share the same ecosystem as potential reservoir species and spillover hosts.

Longitudinal surveillance is essential towards understanding how bat coronaviruses are maintained within a species, as well as the occurrence and duration of shedding [109]. Identification of high-risk shedding periods can direct additional surveillance in other species and allow the formulation of preventative mitigation measures by decreasing possible interactions between human, livestock, and bat population. Determining possible increased shedding times can also allow better planning for surveillance studies to avoid sample collection of cross-sectional studies during the lowest shedding periods. This can be readily accomplished with non-destructive sample collection (colony-level fecal, swabs, or fecal collection). In addition, more basic research is also required for neglected species (Table S4), different animal groups (particularly rodents and livestock) [18] to expand surveillance regions and increase sample sizes.

The reliability of nucleic acid surveillance approaches would be much improved with standardized usage of updated, validated assays such as the recently published assay by Holbrook et al. [148], which updated the widely used Watanabe assay. There has also been an increasing shift away from only publishing short sequences to additional characterization of longer extended sequences or genes. This is both beneficial to the quality of research as well as disadvantageous to having basic surveillance data available. Better characterization of African bat coronaviruses will enable classification of more bat coronaviruses and identifying detectable recombination events. However, this requirement also hampers the frequency of newly published surveillance studies due to escalating costs and sequencing challenges leading to gaps of understanding and unreported diversity among different animal populations. A lack of such standardized approaches also results in technically challenging troubleshooting to be performed in resource-limited laboratories.

Moreover, the cost of fieldwork and sample collection in often remote regions of African countries, as well as the follow-up sample analyses, can be very high, with very little remaining for additional sequencing. Researchers should also be encouraged to publish data on the absence of coronavirus detections to assess species or regions of lower risk. Though not ideal, unpublished nucleic acid surveillance data can also be submitted to NCBI with all relevant collection data. As of August 2020, the user-friendly Database of Bat-associated Viruses (DBatVir) repository contained over 4600 bat coronavirus entries globally [149]. This repository is updated bimonthly, and accessing such a centralized source for bat coronavirus surveillance data (both published and unpublished) will allow for a more comprehensive comparison of detected viruses, assessment of surveillance coverage, and highlight areas where research is required.

We propose that surveillance studies publishing short sequences be bolstered by shifting from detecting viral presence alone to investigating questions concerning the epidemiology and maintenance of coronaviruses in selected populations of different species (Table 5). Bat surveillance in a specific region can be initiated, though it is important that surveillance of other species sharing the same ecological niche be done either concurrently or followed as soon as possible, including potential spillover hosts. Sampling of other animal groups and assessing anthropological and human behavioral risks should be included in the planning and implementation phase. Communities must be at the center of studies to understand societal and cultural issues. Initial surveillance at preselected sites may only provide an overall estimation of animal host species present (bat and non-bat), host movement patterns, and viral excretion, allowing informative planning decisions to be made for proper longitudinal surveillance appropriate sites. Surveillance using short nucleic acid sequences from an updated assay is thus used to identify diversity and monitor changing excretion fluctuations of viruses in populations over time—either seasonally or based on a predetermined time frame (e.g., monthly). This would allow surveillance of both the presence and diversity of coronaviruses among bats and other sampled wildlife/domestic animals and investigate factors involved in viral maintenance with the collection of ecological data. Additionally, such data can be used for a basic assessment of risk regarding potential opportunities for spillover.

Further research is required to characterize detected coronaviruses, including recovery of complete genomes, incorporation of serological studies among bat populations and spillover hosts, or determination of host ranges and zoonotic potential with pathogenesis studies. Issues of cost or technical challenge may be overcome by collaborating with international institutions. In-country expertise and capacity building are essential to build sustainable surveillance programs and require an interdisciplinary approach.

## 7. Conclusions

Surveillance of coronaviruses in wildlife and potential spillover hosts is complicated with logistical, technical, and practical challenges. Proper biosurveillance requires detailed planning ahead of time with well-formulated research questions [150] and essential resources such as highly skilled staff, funding, and operating within ethical and regulatory requirements. Availability of research tools such as appropriate diagnostic assays, standardized protocols, and correct species (specifically related to wildlife) identification is paramount. Studies based on nucleic acid detection have been more commonly used, given the lack of suitable or validated serological assays. The development of such assays is further complicated with issues concerning coronavirus culture in vitro and stringent biosafety Level III conditions. The latter limits research to only a few groups when additional characterization, pathogenicity investigations, and determination of the zoonotic potential of newly discovered bat, rodent, and wildlife coronaviruses is needed. The development of recombinant proteins for serological assays and reverse-genetics systems for coronavirus rescue, though technically complex, are some of the only available options at present.

Much of the coronavirus biosurveillance studies reported, particularly in wildlife, has been reactive to outbreaks/newly emerging viruses and very opportunistic. The current coronavirus research identified many coronavirus host species among bats and rodents and provided novel insights into the possible evolutionary origins of some human coronaviruses [25,35,54]. Moreover, specific groups of coronaviruses have been identified for further research due to lack of characterization and high coronavirus diversity among abundant host populations with opportunities for human contact. The studies mainly provided “snap-shots” of diverse coronaviruses among different species, time points, and geographical locations. Such approaches do not allow long-term monitoring of these viruses in host species toward understanding the factors involved in viral maintenance, nor does it provide cues for interpreting increased risk of spillover. Systematic longitudinal investigations of both natural and potential spillover hosts are needed. Additional layers of investigation must include studying human behavior and anthropological influences and the roles of virus/host interactions, pathogenicity, and the natural ecology of the virus. Investigations of coronavirus diversity among other wildlife (particularly rodents) and livestock are at infancy, with much still unknown. As a result, the future of coronavirus research in African has many topics to cover and will expand continent-wide, requiring an interdisciplinary collaborative approach and significant resource investment.

## Figures and Tables

**Table 1:** Selection and classification criteria of studies included in the review.

**Search criteria:**	Google scholar searches with keywords: “bat, bats, fruit bats, insectivorous bats, animal, mammal, livestock, domestic, domesticated, wildlife, coronavirus, coronaviruses, detections, Africa, Sub-Saharan, Southern Africa, Eastern Africa, nucleic acid, molecular detection, serology, serological, surveillance, survey” were used to search for peer-reviewed publications documenting surveys for coronaviruses in mammals from Africa (mainland Africa as well as islands associated with Africa such as Madagascar, Reunion Island, Seychelles).
**Selection criteria:**	For a suitably thorough synopsis of the findings, publications were limited to research available until the end of December 2020 and excluded dissertations, theses, or non-peer-reviewed publications. Sequences included in phylogenetic analyses in this review also excluded sequences from dissertations, theses, or unpublished sequences on GenBank that are not linked to available publications. However, PREDICT surveillance data (‘PREDICT 1 and 2 surveillance and test data’) linked to a 2017 publication was accessed online from Healthmap.org [56] and included both surveillance among bats and other wildlife and livestock.
**Criteria for ‘primary surveillance reports’:**	Reports containing a description of the collection and testing of samples from animals for coronavirus surveillance. For bat surveillance, we focused on surveillance strategies using nucleic acid detection methodologies such as family-wide consensus PCR analysis or unbiased high throughput metagenomic sequencing. This includes re-testing samples from an earlier report with a different assay and reporting additional coronaviruses detected. Primary surveillance reports may contain varying levels of characterization for detected viruses. We expanded this criterion for livestock and non-bat wildlife to include both nucleic acid and serological surveillance.
**Criteria for ‘secondary characterization reports’:**	Refers specifically to studies based on a primary surveillance report that does not describe new sample collection but a detailed characterization of viral sequences identified in a previous publication or more in-depth analysis of data obtained from primary surveillance reports.

**Table 2:** Bat coronavirus surveillance performed in Africa, per country.

Country (3 Letter Country Code)	References [Primary Surveillance]/(Characterization Report) *
Cameroon	[30,34]
Central African Republic (CAF)	[45]
Democratic Republic of the Congo (DRC)	[30]
Egypt (EGY)	[27]
Gabon (GAB)	[30,40,45]
Ghana (GHA)	[37,44,46]
Guinea (GIN)	[39]
Kenya (KEN)	[19,26,29]/([57])
Madagascar (MDG)	[38,47]
Mauritius (MUS)	[38]
Mayotte (MYT)	[38]
Morocco (MAR)	[38]
Mozambique (MOZ)	[38]
Nigeria (NGA)	[28,41]
Republic of the Congo (COG)	[30,45]
Reunion Island (REU)	[38]
Rwanda (RWA)	[30,35,36]/([60])
Senegal (SEN)	[45]
Seychelles (SYC)	[38]
South Africa (RSA)	[32,42,43]/([58])
Tanzania (TZA)	[30]/([60])
Tunisia (TUN)	[33]
Uganda (UGA)	[30]/([59,60])
Zimbabwe (ZWE)	[31]

* References in square brackets indicate primary surveillance reports; Round brackets refer to ‘secondary characterization reports’.

**Table 3:** Coronavirus detections according bat host taxonomy.

Bat Families Tested	Number of Species	Species Tested	Bat Species Positive	Number of Individuals Tested Per Family *	Positive Individuals ^#^
Pteropodidae	44	22	14	10,851	881 (8.1%)
Hipposideridae	21	10	8	8563	257 (3%)
Molossidae	44	16	8	2144	286 (13.3%)
Miniopteridae	22	12	5	1464	120 (8.2%)
Vespertilionidae	114	37	9	918	41 (4.5%)
Rhinolophidae	38	14	9	728	68 (9.3%)
Emballonuridae	11	4	0	678	0
Nycteridae	15	6	3	299	51 (17.1%)
Rhinonycteridae	6	3	2	250	74 (29.6%)
Megadermatidae	2	2	1	25	3 (12%)
Rhinopomatidae	3	1	0	1	0
Myzopodidae	2	0	0	0	-
Cistugonidae	2	0	0	0	-
Totals	324	127	59	25,921	1779 (6.9%)

* Counts for number of individuals tested reflect individuals from publications reporting total individuals tested per species per country, or total positive individuals in reports where total sampled are not provided. These counts exclude 1966 bats tested in [29,41] from which species totals were not provided, and studies testing colony-level fecal samples [28,31]. ^#^ Approximate number of positives from Table S5.

**Table 4:** Summary of animals (non-bat) tested for coronavirus nucleic acids.

Animals Groups	Birds ^1^ and Poultry/ Other Fowl	Carivores ^2^	Cattle/ Buffalo ^3^	Dogs ^4^	Goats/ Sheep ^4^	Non-Human Primates	Pangolins ^5^	Rodents/ Shrews	Swine ^4^	Ungulates ^7^	Other ^6^	Grand Total
Cameroon	-	**67**	-	-	-	3475	79	**4653**	-	144	16	8434
DR Congo	7	6	10	-	16	1574	3	1848	1	15	2	3482
Ethiopia	-	-	-	-	-	454	-	-	-	-	-	454
Gabon	1	11	-	-	-	82	18	1141	-	548	37	1838
Ghana	-	-	1230	-	2194	496	-	532	716	108	-	5276
Guinea	-	-	-	6	321	-	-	904	8	-	-	1239
Ivory Coast	12	-	-	-	-	**59**	-	293	-	-	-	364
Kenya	-	-	-	-	-	334	-	369	-	514	-	1217
Liberia	-	-	-	-	-	-	-	205	-	-	-	205
Republic of Congo	-	2	-	-	-	352	-	**461**	-	14	-	829
Rwanda	-	-	-	-	-	762	-	**708**	-	-	-	1470
Senegal	-	-	-	-	-	253	-	**263**	-	-	-	516
Sierra Leone	-	5	-	318	938	15	-	369	1012	-	-	2657
South Sudan	-	-	-	-	-	-	-	46	-	-	-	46
Tanzania	-	8	53	120	105	444	-	**1513**	95	**39**	1	2378
Uganda	-	-	-	-	13	1238	-	**762**	1	83	-	2097
Grand Total	20	99	1293	444	3587	9538	100	14,067	1833	1465	56	32,502
Coronavirus nucleic acid	-	1	-	-		14	-	13	-	1	-	29

^1^ Unspecified; ^2^ carnivores (genets, mongoose, and civets; domestic cats); ^3^ domestic and African buffalo; ^4^ domestic; ^5^ tree and long-tailed pangolins; ^6^ ungulates (including camels, duikers, and antelope among others); ^7^ ’other’ (reptiles, snakes, tortoise, hyraxes, and elephants). For species information review [56]. Numbers shaded in bold indicate positive detections from an animal group and country. No recorded surveillance is indicated with a ‘-‘.

**Table 5:** Framework for activity planning when implementing coronavirus surveillance in bat populations, other wildlife species, domesticated animals, and impacted human settlements.

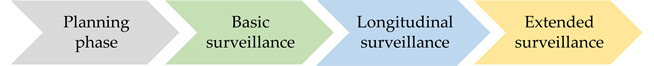	
Consideration	Activity
Formulate a strong research question around the aim of the research to be conducted.	Scope of the surveillance—only coronaviruses or broader surveillance. What will the primary focus of the project be? Assessment of risk for settlements near known colonies? Review the literature and determine important species to target.
Assemble an interdisciplinary team	Collaborate with experts in virology, taxonomists, field biologists, veterinarians, ecologists, specific community leaders, social sciences, and policy-makers. A large interdisciplinary team is essential for accurate long-term surveillance.
Identify high-risk species or animal populations based on a predetermined research question	As a starting point, collaborations can assist in identifying accessible locations of interests, such as specific roosts (day or maternity roosts, etc.) for bat host species considered higher risk (from literature). The roosts can be assessed for population presence over time to enable longitudinal surveillance planning. The region must be assessed for nearby human settlements and the occurrence of animals (farmed, free-roaming, or other wildlife).
Perform initial surveillance targeting either large roosts or multiple smaller roosts	Assess viral presence and diversity with once-off or seasonal surveillance (statistically significant). Population-level sampling of excreted samples such as fecal collection (beneath roosting bats) is simple and non-invasive. Proper species identification should be conducted with both barcodes and morphological identification.
Nucleic acid testing with a suitable assay	Review the literature and use a recently updated assay to ensure detection of all available diversity. Test the assay sensitivity for comparisons. Based on the scope of the project and resource conservation—consider a specific or randomly primed approach.
Plan longitudinal surveillance (duration, types of samples collected, measurement, and ecological data collection). Plan to survey animal species in the region preferably concurrently or sequentially following bat surveillance.	Based on initial findings, plan for longitudinal surveillance according to specified intervals (based on bat presence at roosts or species movements): seasonal or periodic (monthly). Sampling must occur across different reproductive stages. Surveillance can be done at the population-level (overall) and individual-level (to determine demographics of infection prevalence).
Serological surveillance	Review options for serological assays (commercial or developed assays). Collaboration with experts may be critical. Serological testing (bats, non-bat animals, and humans) is important to understand coronavirus antibody responses, duration of protection, and exposure—optimize suitable assays.
Viral characterization	Recover complete genomes of selected viruses for classification and functional studies. Assessing possible zoonotic potential with pathogenesis studies and protein modeling. Collaborate with specialists that can assist and help develop local capacity.
Investigate human-animal interactions	Perform observational and behavioural studies to assess human-wildlife-livestock interactions.

## Supplementary Materials

The following are available online at https://www.mdpi.com/article/10.3390/v13050936/s1, Figure S1: complete Bayesian BEAST phylogeny of African alphacoronaviruses, Figure S2: complete Bayesian BEAST phylogeny of African betacoronaviruses, Table S1: overview of bat surveillance nucleic acid detection studies, Table S2: molecular methodologies employed by the bat surveillance nucleic acid detection studies, Table S3: summary of bat host species tested for coronavirus RNA and positive species reported, Table S4: coronavirus surveillance performed per bat host species, Table S5: bat species from which coronaviruses have been reported (positive species).

## Appendix A

### Details of Phylogenetics

Short sequence lengths may hamper the resolution of a phylogeny, resulting in poor support for certain clades. The phylogenies in Figures S1 and S2 (and Figure 5 and Figure 6) were constructed to include as many African bat coronavirus sequences as possible while still allowing for sequence lengths that would yield well-supported clades. Therefore, sequences that would have resulted in alignments of less than 200 nucleotides were omitted with final lengths between 260 and 294 nucleotides, respectively. For simplicity, all sequence names were converted to the standardized convention with the modification of listing unique sequence identifiers last. Sequences were obtained from Genbank (NCBI) by searching the accession numbers listed in the publications identified as described in Table 1, or manually searching for the publication title. The accession numbers of all sequences included are provided in the phylogenetic trees. Sequence alignments and editing were performed with ClustalW in Bioedit [151]. Maximum clade credibility trees were constructed using suggested models selected from jModelTest2.org [152]. Phylogenetic analyses were performed with Bayesian phylogenetics using BEAST v. 1.10 using the general time-reversible model (GTR) plus invariant sites and gamma distribution substitution model [153]. The CIPRES Science Gateway was used to run computationally expensive analyses such as alignments, jmodeltest, and BEAST [154]. The Bayesian MCMC chains of the alphacoronavirus phylogeny was set to 20,000,000 states, sampling every 2000 steps, and the betacoronavirus phylogeny was set at 25,000,000 states (sampling every 2500 steps). Final trees were calculated from the 9000 generated trees after discarding the first 10% as burn-in. Trees were viewed and edited in Figtree v1.4.2.
