# Supplementary material for: Overview of Bat and Wildlife Coronavirus Surveillance in Africa: A Framework for Global Investigations
Source: Viruses. 2021 May 18;13(5):936. doi: 10.3390/v13050936 (PMC8158508; doi:10.3390/v13050936)
Supplement: Supplementary file 1 [file viruses-13-00936-s001.zip › viruses-1193441-supplementary.pdf]

Table S1: Study overviews						
Report	Country:	Time frame of samples collected:	Type of study and sampling strategy (opportunistic/ archival/ systematic/ repeated sampling):	Sample types collected (collection storage method): *dry = not in a storage or transport buffer	Sample types shown positive for coronaviruses	*Destructive (vouchers collected) or non-destructive (catch-and release) sampling:*
Tong et al. 2009 [26]	Kenya	July-August 2006	Sampling of 17 identified roosts over 2 months among the southen regions of Kenya; sampling not repeated in the study duration.	Fecal swabs, oral swabs; blood; tissues (collected dry with cold-chain)	Fecal swabs	Destructive; euthanized all bats
Pfefferle et al. 2009 [37]	Ghana	February 2008	Surveillance of bats present at 4 sites near a lake habitat, rural area and university. Diverse insectivorous bats was targeted, and a large urban roosting site of frugivorous bats. Sampling not repeated in the study duration.	Fecal material from individual bats and fecal samples collected with plastic foil under trees occupied by Eidolon helvum bats (1–4 fecal pellets or swabs suspended in RNAlater, Applied Biosystems)	Fecal material	Non-destructive
Quan et al. 2010 [41]	Nigeria	June 2008	Sampling of bats roosting in caves around human dwellings, that are frequented by people; sampling not repeated in the study duration.	Gastrointestinal tract specimens (collected dry with cold-chain)	Gastrointestinal tract specimens	Destructive
Geldenhuys et al. 2013 [42]	South Africa	2005-2011 (archival- 7 years)	Opportinistic testing of approppropriate sample types in an archival biobank for initial surveillance of bats previously collected to determine if coronaviruses were present.	Fecal material and rectal specimens (archival)	Fecal material and rectal specimens	Combination - samples were available and not specifically collected for this surveillance, though bats were entered in museum collections
Ithete et al. 2013 [43]	South Africa	2010-2012	Sampling of 4 known roosts among the western and eastern coastal regions of South Africa; not specified if or how many times repeated sampling at same locations	Fecal material (suspended in RNAlater, Life Technologies)	Fecal material	Combination; bats were entered in museum collections
Annan et al. 2013 [44]	Ghana (also surveyed in Germany, the Netherlands, Romania, and Ukraine)	2009-2011	Sampling of selected species as a second surveillance study in Ghana. A total of 7 locations were surveyed across southern regions of the country. Not specified if or how many times repeated sampling at same locations.	Fecal material (suspended in RNAlater Stabilization Reagent, Qiagen)	Fecal material	Non-destructive
Maganga et al. 2014 [45]	Republic of Congo, Gabon, Central African Republic (CAR) and Senegal	2005-2008; Republic of Congo (2005 and 2006); Gabon (2005, 2006, 2009 and 2010); CAR (2008 and 2009); Senegal (2006)	Sampling aimed to investigate determinants affecting viral richness in bats sampled in Central and West Africa. Bats were sampled at multiple locations, at once-off sampling (no repeat sampling of the same localities).	Collected organs (not specified)	Intestine, lung	Destructive
Corman et al. 2015 [46]	Ghana	2009-2011	Sampling building on the collection from Annan et al. 2013; Focused surveillance for viruses related to human coronavirus 229E, with characterization of full genomes.	Fecal material in RNAlater (suspended in RNAlater Stabilization Reagent, Qiagen)	Fecal material	Non-destructive
Razanajatovo et al. 2015 [47]	Madagascar	2007-2011	Sampling at 8 sites in Madagascar, in known accessible roosts (trees and caves) where bats are frequently hunted as bushmeat. Some sites sampled more than once.	Rectal and throat swabs in viral transport media (unspecified)	Rectal swabs	Non-destructive

Shehata et al. 2016 [27]	Egypt (and Lebanon)	February 2013–April 2015	Surveillance in bats collected from Egypt and Lebanon. Not specified if or how many times repeated sampling at same locations. Caves were in proximity to human-inhabited area but not in proximity to camels.	Oral and rectal swabs as well as blood; lung and liver from select individuals. (not specified)	Lung, liver, rectal and oral swabs	Combination; 821 sampled with 72 euthanized
Leopardi et al. 2016 [28]	Nigeria	2011 (sampled twice in 2011)	Twice-sampled; surveillance for coronaviruses of an urban colony of bats that are regularly consumed as bushmeat.	Colony-level fecal (not specified)	Colony-level fecal	Non-destructive
Tao et al. 2017 [19]	Kenya	2007-2010 (4 years)	Sampling of 30 known roosts among the southen regions of Kenya; not specified if or how many times repeated sampling at same locations	Fecal swabs & oral swabs (suspended in PBS)	Fecal swabs & oral swabs	Non-destructive
Waruhiu et al. 2017 [29]	Kenya	2012-2015 (November to November over 4 years)	Surveillance for bat viruses - survey for various DNA and RNA viruses (astro-, adeno-, calici-, corona-, flavi-, filo-, paramyxo-, polyoma- and rotaviruses); 48 sites mostly southern localities and included diverse roosts all with high level of human interactions (caves, trees, inhabited and abandoned buildings like offices and homes). Not specified if or how many times repeated sampling at same locations.	Fecal material (1 fecal pellet per tube in RNAlater Stabilization Reagent, Qiagen). Fecal material collected overnight on sheets.	Fecal material	Non-destructive
Anthony et al. 2017a [30]	Central Africa: Cameroon, Gabon, Democratic Republic of Congo, Republic of Congo, Rwanda, Tanzania, Uganda (also in the study Latin America: Peru, Bolivia, Brazil, Mexico, and Asia: Bangladesh, Cambodia, China, Indonesia, Laos, Malaysia, Nepal, Thailand, Viet Nam).	5 years	Global coronavirus assessment in bat species investigating 20 countries in 3 continents (Latin America, Africa, and Asia) over 5 years. The aim of the study was to identify diversity and factors driving this diversity. Samples were collected at 'high risk' interfaces (deforestation and agricultural expansion, around human dwellings; places of ecotourism, markets, wildlife restaurants and farms, where occupational exposure was likely), where direct or indirect contact with humans might promote zoonotic viral transmission.	Swabs (e.g. oral, urine, rectal), fluids (e.g. saliva), and tissues were collected into either NucliSensVR Lysis Buffer, BioMe´rieux, Inc. or viral transport media (unspecified) with cold-chain	Rectal swabs & oral swabs	Combination - (predominantly non-descructive with some destructive sampling)
Bourgarel et al. 2018 [31]	Zimbabwe	June 2016 and February 2017 (10 months)	Investigation of fecal samples at two cave sites regularly visited by local people to collect bat guano used as fertiliser for viral RNA (coronaviruses and paramyxoviruses). Each site was visited twice during the sampling period.	Colony-level fecal samples collected on 5 plastic sheets per cave (overnight inside the caves), pooled faeces were mixed with 6 ml of in-house RNA stabilisation solution, Pol Scientific.	Colony-level fecal	Non-destructive

Geldenhuys et al. 2018 [32]	South Africa	2007-2015 (8 years)	A metagenomic study targeting the gastrointestinal virome of the *Neormicia* genus in North Eastern and Eastern coastal regions of South Africa; archival samples (n=58) were utilised with a unbaised high-throughput sequencing approach and followed up with PCR analyses of remaining samples (n=42) to determine specific host species identified to harbour specific viruses; repeat sampling at certain locations	Gastrointestinal samples such as fecal pellets and rectal and intestinal specimens (collected dry with cold-chain)	fecal mateiral and intestinal specimens	Combination - samples were available and not specifically collected for this surveillance, though bats were entered in museum collections
Ar Gouilh et al., 2018 [33]	Tunisia and Morocco	2012	The study surveyed bats for coronaviruses predominantly in France and Spain (including different regions to include varying climates), with some opportunisitic sampling in Morocco and Tunisia. Focus was directed towards regions of large bat diversity inclusing maternity colonies. Not specified if or how many times repeated sampling at same locations.	Colony-level fecal as well as fecal material from individual bats (viral transport medium with antibiotic supplements or RNA later from Ambion)	Fecal material	Non-destructive
Yinda et al. 2018 [34]	Cameroon	December 2013 and May 2014	Metagenomics study using samples collected from 3 locations with the South West Region of Cameroon; no repeat sampling	Fecal material (collected dry with cold-chain)	Fecal material	Non-destructive
Markotter et al. 2019 [35]	Rwanda	2008	Surveillance of Rwandan bat species for the presence of coronaviruses (and paramyxoviruses); not repeated in the study duration	Fecal material, rectal and intestinal samples (suspended in RNAlater Stabilization Reagent, Qiagen)	Fecal material	Combination; bats that died during processing were taken as vouchers and tissues harvested
Nziza et al. 2019 [36]	Rwanda	2010-2014	Surveillance for bat viruses - survey for various RNA viruses (corona-, alpha-, arena-, bunya-, filo-, flavi-, hanta-, influenza-, paramyx-, lenti- and rhabdoviruses.); 25 sites selected and include urban and rural sites characterized by a human–wildlife interface. Frequency of repeat sampling not specified. Part of USAID Emerging Pandemic Threats PREDICT project -reported in Anthony et al. 2017.	Rectal & oral swabs collected into either BD Universal Viral TransportTM medium and/or NucliSENS Lysis Buffer	Rectal swabs & oral swabs	Non-destructive
Joffrin et al. 2020 [38]	Mozambique, Madagascar, Mauritius, Mayotte, Reunion Island and Seychelles	Mozambique: Feb-May 2015; Mayotte: Nov-Dec 2014; Reunion Island: Feb 2015; Seychelles: Feb-Mar 2014; Mauritius: Nov 2012; Madagascar: Oct-Nov 2014; Jan 2018	Sampling of the diverse and often isolated species on islands in the Western Indian Ocean. Testing of historical samples combined with more recent sampling events; sampling not neccesarily repeated in the study duration.	Organs (Intestine and lung) collected before 2014 - stored dry. Feces, rectal, and oral swabs after 2014 were collected in 1mL brain heart infusion medium (Conda, Spain) supplemented with antifungals and antibacterial agents. All were stored in liquid nitrogen in the field.	Intestines, feces and rectal swabs	Combination - some samples were previously collected and included in the surveillance, others include non-destructive fecal collection and swabs.
Lacroix et al. 2020 [39]	Guinea	Feb 2016 - Jan 2017	Surveillance of samples previously targeted for ebolavirus and tested for coronaviruses from 7 sites; sampling not repeated in the study duration	Rectal and oral swabs an opportunistic fecal (in RNALater, ambient temperature)	Rectal and oral swabs as well as feces	Non-destructive

Maganga et al. 2020 [40]	Gabon	2009-2015	Surveillance of 6 bat caves in Northern Gabon along with non-human primates, rodents and other wildlife (bushmeat) species in the area. Sampling not repeated in the study duration	Intestines (dry) and feces (RNALater) (frozen in liquid nitrogen)	Intestines	Combination - bushmeat, bat and rodent vouchers and collection of fecal samples from bats and wildlife.

Table S2: summaries of study methodologies

Report	Methods summary	RNA extraction kit	Random / specific primers for cDNA	Assay enzyme or kit used	Assay primers used/based on	Gene region amplified by surveillance assay (approximate nucleotide position vs. NC_004718.3 SARS coronavirus Tor2)	Surveillance region	Surveillance amplicon size	Sequence accession numbers in manuscript:	Sequence naming functional	Performed further characterization (molecular):	Specific sample processing describedbefore extraction of nucleic acids	Isolation attempted
Tong et al. 2009 [26]	Nested RT-PCR was performed with two sets of coronavirus primers designed in the study. Primers are provided.	QIAMP mini viral spin kit (QIAGEN);	Specific	Superscript 3 One-step kit with platinum taq (Invitrogen)	Tong et al. 2009 (developed)	Segment of the RNA-dependent RNA polymerase gene (Nested: 15213-15412 nt)	Universal	121	Not present; GQ920800-GQ920838	*Not informative in phylogeny as it does not allow identification of host associated with sequence; eg. BtKY30 (also not listed anywhere in association with a host)*	Not in present manuscript; Tao et al. 2012 desceibesadditional characterization.	Not specified	Not attempted/ described
Pfefferle et al. 2009 [37]	Nested RT-PCR was performed according to assay and modified primers from a selected reference.	Viral RNA minikit (QIAGEN);	Specific	Not-specfied [One-step RT-PCR kit (Qiagen) with Platinum Taq (Invitrogen) according to reference]	de Souza Luna et al. 2007	Segment of the RNA-dependent RNA polymerase gene (Nested: 15210-15664 nt)	Universal	450	FJ710043-FJ710056	*Yes - e.g. BtCoV/Hip/GhanaBoo/348/2008 allows identification of information such as host, country, year etc. associated with the sequence*	Specific primers were designed for sequencing longer fragments of the RdRp gene of representative viruses (817 bp of detected alphacoronaviruses and 1,221-bp of betacoronaviruses) as well as nucleoprotien genes. Also performed end-point dilution experiments with the nested pan-CoV RT-PCR (de Souza Luna et al. 2007) to determine viral load. Lastly performed MRCA analyses with available sequence fragments.	Not specified	Not attempted/ described
Quan et al. 2010 [41]	Hemi-nested RT-PCR was performed according to primers designed in the study. Primers are provided.	Not-specified	Specific	cDNA with SuperScript 3 kit (Invitrogen) and Hot-Star polymerase (Qiagen)	Quan et al. 2010	Segment of the RNA-dependent RNA polymerase gene (Nested: 18386-18717 nt)	Non-Universal	400	HQ166910	Somewhat; Zaria bat coronavirus is named for the location in Nigeria and denotes the host, but does not associate a specific species to the virus or year	Sequenced the complete genome with unbiased high-throughput pyrosequencing	Not specified	Not attempted/ described
Geldenhuys et al. 2013 [42]	Genus-specific heminested RT-PCR assay designed in this study as performed. Primers available on request.	TRIzol (Invitrogen);	Specific	Not-specified	Geldenhuys et al. 2013 (based on primers from Woo et al. 2005)	Segment of the RNA-dependent RNA polymerase gene (Nested:15279-15655 nt)	Universal	Not specified; 360	Not present; JQ519817-JQ519819	*Yes - e.g. Mops-BtCoV/1364/SA/11 allows identification of information such as host, country, year etc. associated with the sequence*	None	Not specified	Not attempted/ described
Ithete et al. 2013 [43]	Nested RT-PCR was performed according to assay and modified primers from a selected reference.	QIAamp Viral RNA Mini Kit (QIAGEN)	Specific	Not-specified	de Souza Luna et al. 2007	Segment of the RNA-dependent RNA polymerase gene (Nested: 15210-15664 nt)	Universal	450	Not all accessions provided; KC869678, KF843851-KF843862	*Yes - e.g. BtCoV/PML/2011/Neo_zul/RSA/201 allows identification of information such as host, country, year etc. associated with the sequence*	Extended sequenced regions to 819 bp with RdRp grouping units PCRs designed by Drexler et al. 2010. Corman et al. 2014 described full genome sequencing and characterization of the detected betacoronavirus.	Not specified	Not attempted/ described
Annan et al. 2013 [44]	Nested RT-PCR was performed according to assay and modified primers from a selected reference.	Viral RNA minikit (QIAGEN);	Specific	Not-specfied [One-step RT-PCR kit (Qiagen) with Platinum Taq (Invitrogen) according to reference]	de Souza Luna et al. 2007	Segment of the RNA-dependent RNA polymerase gene (Nested: 15210-15664 nt)	Universal	450	JX899382–JX899384	*Yes - e.g. BtCoV/PKW2E-F82/Nyc_spec/GHA/2011 allows identification of iinformation such as host, country, year etc. associated with the sequence*	Real-time RT-PCR designed to permit sensitive and quantitative detection of lineage C betacoronaviruses; extended sequenced regions to 819 bp with RdRp grouping units PCRs designed by Drexler et al. 2010	Not specified	Not attempted/ described
Maganga et al. 2014 [45]	Nested RT-PCR was performed according to assay and modified primers from a selected reference.	Not-specified	Not-specified	Not-specified	de Souza Luna et al. 2007	Segment of the RNA-dependent RNA polymerase gene (Nested: 15210-15664 nt)	Universal	Not specified	JX174638-JX174640; JX174641-JX174642	*Not informative; no information regarding virus or host species in name, only location, unique identifier and year eg. CAR/P 31/2009*	None	Not specified	Not attempted/ described

Corman et al. 2015 [46]	Developed ans used a real-time RT-PCR assay for the detection of bat coronaviruses genetically rellated to HCoV-229E. Primer and probed are provided.	MagNA Pure 96 system (Roche)	Specific	Superscript 3 RT-PCR kit (Invitrogen)	Corman et al. 2015	Segment of the RNA-dependent RNA polymerase gene (real time: 13947-14137 nt of NC_002645 HCoV229E)	Non-Universal	Not specified	KT253259 to KT253323	*Yes - e.g. BtCoV/BUO2E-F160/Hip cf. rub/GHA/2011 allows identification of information such as host, country, year etc. associated with the sequence*	Obtained extended sequenced regions to 816 bp with RdRp grouping units PCRs designed by Drexler et al. 2010 and full genomes sequences for 4 bat 229E-related clades	Not specified	Not attempted/ described
Razanajatovo et al. 2015 [47]	Nested RT-PCR was performed with primers from a reference, an additional hemi-nested step was incorporated with primers designed in this study. Primers are provided.	QIAamp Viral RNA minikit (QIAGEN);	Random	cDNA with M-MLV Reverse transriptase (Invitrogen)	Razanajatovo et al. 2015 (based on Poon et al. 2005)	Segment of the RNA-dependent RNA polymerase gene (Nested: 15216-15656 nt)	Universal	329	KF859758-KF859771; KP696741-KP696747	*Yes - e.g. BatCoV074F\Pteropus rufus\KP696745\2011 allows identification of information such as host, year etc. associated with the sequence (lacks location)*	Extended sequenced regions of the RdRp gene to 1086 bp with strain-specific primers	Not specified	Not attempted/ described
Shehata et al. 2016 [27]	Nested RT-PCR was performed according to assay and primers described in this study. Primers are provided in technical appendix.	QIAamp viral RNA minikit (QIAGEN)	Specific	One-step RT-PCR kit (Qiagen)	Shehata et al. 2016	Segment of the RNA-dependent RNA polymerase gene (Nested: 15266-15655 nt)	Universal	440	Not all accessions provided; KT346237-KT346243, KT581595, KT581600, KT581602	*Yes - e.g. Rousettus/Egypt/NRC-HKU-B81 allows identification of information such as host and country etc. associated with the sequence*	MERS-specific envelope-gene quantitative RT-PCR was performed as well as serological testing of 370 serum samples. All negative. PCR designed specifically for this study was used for amplifying N gene sequences of HKU9-related viruses (primers provided in technical appendix).	Not specified	Not attempted/ described
Leopardi et al. 2016 [28]	Nested RT-PCR was performed according to assay and modified primers from a selected reference (modifications not provided)	Nucleospin RNA II kit (Macherey - Nagel)	Specific	Superscript 3 One-step RT-PCR kit (Invitrogen)	de Souza Luna et al. 2007	Segment of the RNA-dependent RNA polymerase gene (Nested: 15210-15664 nt)	Universal	398	KU131210 to KU131215	*Yes - e.g. BtCoV/KU131213/59/Eidolon_helvum/Nigeria/2011 allows identification of information such as host, country, year etc. associated with the sequence*	Continued with sequenced region extension using targeted pathogen genome amplification with Sanger and next generation sequencing (MiSeq-Illumina) approaches (approximatley 800 and 2000 bp)	Not specified	Not attempted/ described
Tao et al. 2017 [19]	Nested RT-PCR was performed with Pan bat CoV RT-PCR primers from a given reference (with no modifications given)	QIAMP mini viral spin kit (QIAGEN)	Specific	Superscript 3 One-step RT-PCR kit (Invitrogen)	Tong et al. 2009	Segment of the RNA-dependent RNA polymerase gene (Nested: 15213-15412 nt)	Universal	400	Not all sequences are submitted to Genbank; KY073744 to KY073748	*Yes - e.g BtKYNL63-9b Triaenops afer or BtKY237 Rhinolophus hilderbrandtii allows identification of a unique identifier, country and hostassociated with the sequence*	Obtained complete genomes of selected lineages with detailed characterization	Not specified	Not attempted/ described
Waruhiu et al. 2017 [29]	Nested RT-PCR was performed with modified primers from a given reference. Primers are provided in technical appendix.	Pure viral RNA kit (Roche)	Specific	Superscript 3 One-step RT-PCR kit (Invitrogen)	Waruhiu et al. 2017 (based on Watanabe et al. 2010)	Segment of the RNA-dependent RNA polymerase gene (Nested: 15216-15655 nt)	Universal	434	Not present; MH170074-MH170150	*Yes - e.g. BatCoV/BAT2363/Hipposideros caffer/KEN/Meru/2015, allows identification of information such as host, location, year etc. associated with the sequence*	None	Collected fecal was vortexed and undwent clearing centrifugation of which 200µL was used for viral nucliec acid extraction.	Not attempted/ described
Anthony et al. 2017a [30]	Two separate assays for coronavirus detection used prepared cDNA to amplify non-overlapping segments of the target ORF - primers from given references.	Not specified	Not-specified	cDNA with Superscript 3 (Invitrogen)	Quan et al. 2010 and Watanabe et al. 2010	Two different segments of the RNA-dependent RNA polymerase gene (Quan et al. Nested: 18386-18717 nt; Watanabe et al. 15216-15654 nt)	Universal and non-universal	332 and 434	Not present; KX284927-KX286327	*Not informative regarding source of reported sequence (host/location etc) if sequences are named after most similar previously described relative. E.g. sequence named "Kenya bat coronavirus/BtKY56/BtKY55 PREDICT-GVF-CM-ECO06409" refers to a Epomops coronavirus from Cameroon.*	None	Not specified	Not attempted/ described
Bourgarel et al. 2018 [31]	A total of 123 pools were analysed with primers from a given reference.	NucleoSpin® RNA Kit (Macherey-Nagel)	Random	Not described	Chu et al. 2011	Segment of the RNA-dependent RNA polymerase gene (Nested: 15216-15656 nt)	Universal	415	MG000865-MG000872	*Yes - e.g. BtCoV/CR001Mab_Hip.spp_ZIM allows identification of information such as host, country etc. associated with the sequence (lacks year)*	None	Fecal matter (+/-6g) was pooled and underwent twice clearing centrifugation and filtration prior to pelleting with ultracentrifugation (250,000 g for 2.5 hours at 4 °C).	Not attempted/ described

Geldenhuys et al. 2018 [32]	Viral metagenomics was followed up with consensus PCR confirmation using a developed multiplexed genus-specific nested RT-PCR assay. Primers are provided in technical appendix.	Duet RNA/ DNA miniprep plus kit (ZymoResearch)	Random	cDNA with Superscript 3 (Invitrogen)	Metagenomics; Geldenhuys et al. 2018 (based on Geldenhuys et al. 2013)	Segment of the RNA-dependent RNA polymerase gene (Nested: 15388-15655 nt)	Universal	268	MF593268 and MF593271 (coronaviruses)	*Yes - e.g. BatCoV/Neo5038/KZN/RSA/2015 allows identification of information such as host, country, year etc. associated with the sequence*	Extended the alphacoronavirus sequenced region to 600 bp as well as full genome charaterization of the betacoronavirus	Not specified	Not attempted/ described
Ar Gouilh et al., 2018 [33]	Nested RT-PCR was performed with primers from a given reference.	Not specified	Random	cDNA with Superscript 3 (Invitrogen)	Gouilh et al., 2011	Segment of the RNA-dependent RNA polymerase gene (Nested: 15438-15655 nt)	Universal	220	KY423445, KY423485-KY423487	*Yes - e.g. AlphacoV_EP19SPA/TUN_Myomyo/Myopun_2011/2012 allows identification of information such as host, country, year etc. associated with the sequence*	None		Not attempted/ described
Yinda et al. 2018 [34]	Enriched for viral particles using the NetoVIR protocol (Conceicao-Neto et al. 2015) and sequenced on the Illumina HiSeq 2500 platform for 300 cycles.	QIAamp Viral RNA Mini Kit (Qiagen, Hilden, Germany)	-	Whole Transcriptome Amplification Kit, Sigma-Aldrich.	Metagenomics	Complete genomes obtained	Not applicable	metagenomics	MG693168-MG693172	*Yes - e.g. Bat-CoV/900/Eidolon helvum/2014/CMR allows identification of information such as host, country, year etc. associated with the sequence*	Obtained partial/complete genomes with highthroughput sequencing	Prepared 25 pools (1-5 samples per pool) that were homogenized and underwent filtration trhough a series of membrane filters. The filtrate was then treated with an enzyme cocktail of to digest free-floating nucleic acids.	Not attempted/ described
Markotter et al. 2019 [35]	Nested RT-PCR was performed with primers from a given reference.	Duet RNA/ DNA miniprep plus kit (ZymoResearch)	Random	cDNA with Superscript 3 (Invitrogen)	Geldenhuys et al. 2018	Segment of the RNA-dependent RNA polymerase gene (Nested: 15388-15655 nt)	Universal	268	JQ649535-JQ649536	*Yes - e.g. Rh-BtCoV/441/Rwanda/08 and Rh-BtCoV/445/Rwanda/08 allows identification of information such as host, country, year etc. associated with the sequence*	Extended sequenced regions to 820 bp with RdRp grouping units PCRs designed by Drexler et al. 2010	Not specified	Not attempted/ described
Nziza et al. 2019 [36]	Two separate assays for coronavirus detection used prepared cDNA to amplify non-overlapping segments of the target ORF - primers from given references.	QIAamp Viral RNA Mini Kit (QIAGEN)	Not-specified	cDNA with Superscript 3 (Invitrogen)	Quan et al. 2010 and Watanabe et al. 2010	Two different segments of the RNA-dependent RNA polymerase gene (Quan et al. Nested: 18386-18717 nt; Watanabe et al. 15216-15654 nt)	Universal and non-universal	332 and 434	KX286324-KX285828, KX285830, KX285106-KX285108, KX286259, KX285819, KX285821, KX285822, KX285111	*Not informative regarding source of reported sequence (host/location etc) if sequences are named after most similar previously described relative. E.g. sequence named "Kenya bat coronavirus/BtKY56/BtKY55 PREDICT-GVF-CM-ECO06409" refers to a Epomops coronavirus from Cameroon.*	None	Not specified	Not attempted/ described
Joffrin et al. 2020 [38]	Real time assays in combination with conventional assays for phylogeny.	QIAamp Viral RNA mini kit (QIAGEN)	Random	ProtoScript II Reverse Transcriptase and Random Primer 6 (New England BioLabs); ABsolute Blue QPCR Mix low ROX 1X (Thermo Fisher Scientific)	Muradrasoli, et al. 2009 (real time); Poon et al.2005 (conventional PCR)	Segment of the RNA-dependent RNA polymerase gene (Real time corresponding to 15647-15825 nt; conventional assay corresponding to: 15216-15655 nt)	Universal and non-universal	Real time: 179; Conventional assay: 440	MN183146-MN183273	*Yes - e.g. Bat_CoV_FMNH_229303_Mozambique_ Mo.condylurus _2015_MN183182 allows identification of unique identifiers, location and host.*	No, real-time followed by conventional PCR assay based on the assay from Poon et al 2005.	Not specified	Not attempted/ described
Lacroix et al. 2020 [39]	Nested RT-PCR was performed with modified primers from a given reference. Primers are not provided.	NucliSENS EasyMAG platform (BioMérieux, Marcy-l'Etoile).	Random	Reverse Transcription System kit with random primers (Promega); GoTaq Hot Start Master Mix PCR kit (Promega)	Chu et al. 2011; (modified protocol)	Segment of the RNA-dependent RNA polymerase gene (Nested: 15216-15656 nt)	Universal	440	MT586830-MT586867	*Yes - e.g. CCGU33_Ep_gambianus_GN though missing some data, it does allow identification of the host and country*	None	Not specified	Not attempted/ described
Maganga et al. 2020 [40]	Nested RT-PCR surveillance of samples performed according to a published assay, with high throughput sequencing on selected positives	EZ1 RNA tissue mini kit (Qiagen)	Specific	Qiagen One-step RT PCR kit (Qiagen), and Platinum Taq DNA polymerase kit (Life Technologies).	de Souza Luna et al. 2007	Segment of the RNA-dependent RNA polymerase gene (Nested: 15210-15664 nt)	Universal	494	MG963186-MG963189 and MG963191-MG963201	*Yes- e.g . MG963196_09GB0376 Hipposideroscf ruber_2009 allows identification of host and year of detected sequence*	Attempted high throughput MiSeq sequencing of three positive samples. The cDNAs were prepared with SuperScript III (Thermo Fisher Scientific). Size selection was performed followed by library preparation with the NEBNext Ultra RNA Library Prep kit for Illumina.	Nearly 100 mg intestines were pooled according to species and homogenized in PBS using a ball-mill tissue grinder (Geno/Grinder 2000, Spex Centripep). RNALater was removed from feces preserved therein, suspended in PBS, and centrifuged at 1500 rpm for 5 min.	Not attempted/ described

Table S3: summaries of species tested and positive percentages obtained per studies						
Report	Number of bat species (genera) tested in total:	Average number of individuals per species (range of number of individuals):	Number of species below 10 individuals (% of species in study):	Number of species found to harbour coronaviruses (% species positive):	Overall positivity:	Host species identification methods:
Tong et al. 2009 [26]	21 species (14 genera)	10.5 (1-39)	12 (57.1%)	11 species (52.4%)	18.6% (41/221)	Not described
Pfefferle et al. 2009 [37]	10 species (7 genera)	13.7 (1-59)	6 (60%)	1 species (10%)	3.6 % (12/335)	Morhological identification confirmed with mitochondrial cytochrome b gene sequencing
Quan et al. 2010 [41]	6 species (5 genera)	Cannot be determined; totals per species tested are not specified (total of 33 bats tested)	-	1 species (16.7%)	3.0% (1/33)	Not described
Geldenhuys et al. 2013 [42]	29 species (14 genera)	3.8 (1-14)	27 (93.1%)	3 species(10.3%)	2.7% (3/113)	Not described
Ithete et al. 2013 [43]	12 species (10 genera)	5.5 (1-13)	9 (75%)	1 species (8.3%)	8% (5/62)	Experienced chiroptologist used morphological systematics
Annan et al. 2013 [44]	10 species (5 genera)	475.8 (1-3763)	2 (20%)	1 species (10%)	0.97% (46/4758)	Not described
Maganga et al. 2014 [45]	Overall: 15 species (14 genera)	101.6	7 (46.67%)	2 species (13.3%)	0.13% (5/3874)	Field identification by trained field biologist and confirmed with sequencing the mitochondrial cytochrome b gene sequences.
	Gabon: 12 species (11 genera)	162.75 (15-492)	0 species	1 species	0.2% (3/1953)	
	Republic of Congo: 6 species (6 genera)	125 (2-286)	3 (50%)	0 species	0%	
	Senegal: 2 species (2 genera)	16.5 (15-18)	0 species	0 species	0%	
	Central African Republic: 11 species (11 genera)	103 (1-533)	4 species (36.4%)	1 species	0.2% (2/1138)	
Corman et al. 2015 [46]	11 species (7 genera)	189.73 (4-1611)	4 (36.4%)	2 species (18.2%)	3.9% (81/2087)	Morphologically identified in the field
Razanajatovo et al. 2015 [47]	3 species (3 genera)	104.3* (76-141)	0 species	2 species (66.7%)	4.5% (14/313*)	Identified using morphological features by field trained team (ecologist and veterinarian)
Shehata et al. 2016 [27]	3 species (3 genera)	123.3 (31-257)	0 species	2 species (66.7%)	5.1% (19/370)	Not described
Leopardi et al. 2016 [28]	1 species (1 genus)	Cannot be determined as colony-level fecal samples were collected	-	1 species (100%)	7.6 % (6/79) within the sampled fecal material	Not described
Tao et al. 2017 [19]	27 species (17 genera)	76.3 (2-397)	5 (18.5%)	18 species (66.7%)	11.7% (240/2050)	Trained field biologist and confirmed with Cytochrome B sequencing

Waruhiu et al. 2017 [29]	22 species (16 genera)	Cannot determine average number of individuals sampled or ranges of most to least sampled; Total fecal collected: 3334, and only 2014 tested for coronaviruses. No totals of bat individuals/species tested are not specified; totals are given tested per site (all species), with positive species and individuals indicated.	-	9 species (40.9.7%)	4% (80/2014)	Experienced chiroptologist and confirmed with Cytochrome B sequencing
Anthony et al. 2017a [30]	Totals positive and numbers per species are not indicated in Anthony et al. 2017a; sample numbers			34 species	9.4% (1097/11704)	Lowest taxonomic order identified (genus and species) and assigned to an age class (adult, subadult, neonate) by the field teams
	Cameroon	31 (1-674)	31 (58.5%)	20 species (37.7%)	7.8% (210/2679)	
	DRC	46 (1-520)	16 (49%)	10 species (30%)	5.5% (84/1520)	
	Gabon	217 (2-859)	4 (36%)	1 species (9%)	0.1% (3/2389)	
	COG	19 (1-148)	26 (70%)	13 species (35%)	8.9% (66/734)	
	Rwanda	41 (1-286)	12 (50%)	11 species (46%)	7.1% (70/990)	
	Tanzania	168 (1-1432)	6 (40%)	7 species (47%)	25.7% (647/2517)	
	Uganda	49 (1-270)	13 (72%)	5 species (28%)	1.9% (17/875)	
Bourgarel et al. 2018 [31]	1 species (1 genus)	Cannot be determined as colony-level fecal samples were collected	-	1 species (100%)	6.5% (8/123)	Cytochrome B sequencing
Geldenhuys et al. 2018 [32]	4 species (1 genus)	10.5 (3-24)	3 (75%)	1 species (25%)	4.8% (2/42)	Experienced taxonomist in collaboration with a museum and confirmed with mitochondrial cytochrome c oxidase subunit I (COI) gene sequencing
Ar Gouilh et al., 2018 [33]	5 species (4 genera)	4.4 (2-8)	5 (100%)	2 species (40%)	16% (4/25)	Morphological identification, acoustic data and confirmed with cytochome B gene sequencing
Yinda et al. 2018 [34]	2 species (2 genera)	43.5 (1-85)	1 (50%)	1 species (50%)	52% of 25 pools	Not described
Markotter et al. 2019 [35]	5 species (5 genera)	20.2 (2-72)	3 (40%)	1 species (20%)	2% (2/101)	Morphological identification and confirmed with cytochome B or cytochrome oxidase one gene sequencing
Nziza et al. 2019 [36]	16 species (14 genera)	31 (1-130)	8 (50%)	8 species (50%)	5.4% (27/503)	Morphological and confirmed with cytochome b (cyt b) or cytochrome oxidase one (COI) gene
Joffrin et al. 2020 [38]	36 species (18 genera)	26.7 (2-94)	12 (33.4%)	13 species (36.1%)	8.7% (88/1013)	Not described - mitochondrial cytochrome b gene sequencing

Lacroix et al. 2020 [39]	14 species (14 genera)	22.8 (1-120)	9 (64.3%)	8 species (57%)	11% (35/319)	Morphological measurements and molecular barcode (cytochrome b) confirmation.
Maganga et al. 2020 [40]	5 species (4 genera)	213 (112-287)	0 species	2 species (40%)	1.41% (15/1066)	Bat and rodent species were identified by trained field biologists.

* specifically refers to rectal swabs

Table S4: Coronaviruses detected per species							
Taxonomic rank and extinct taxa (extinct taxa are indicated in grey text)	Species	Coronavirus detected in species (yes/no)	Number of individuals tested (counting totals of individuals provided in studies and positive individuals in reports where totals are not provided)	Types of coronavirus detected; similar to specified clades (refer to "like" as their grouping within subgenera are not certain)	Countries (all locations tested)	References	Reported as Bushmeat (Reviewed in Mildenstein et al 2016)
FAMILY Pteropodidae		14/44 (22 tested; 22 not tested)	10851				
Genus Eidolon		2/2	3096				
	Eidolon dupreanum	Yes	96	BetaCoV (Nobecovirus)	Madagascar	Razanajatovo et al. 2015	Yes
	Eidolon helvum	Yes	3000	AlphaCoV (Novel); BetaCoV (Nobecovirus)	Cameroon, Kenya, Nigeria, Ghana, Gabon, Senegal, Central African Republic, Tanzania, Rwanda, Republic of the Congo, Democratic Republic of the Congo, Uganda, Guinea	Pfefferle et al., 2009; Maganga et al. 2014; Anthony et al. 2017a,. Tong et al. 2009, Waruhiu et al. 2017, Tao et al. 2017, Leopardi et al. 2016, Yinda et al. 2018, Nziza et al. 2019; Lacroix et al. 2020	Yes
Genus Pteropus		1/8	196				
	Pteropus aldabrensis	-	-				
	Pteropus livingstonii	-	-				Yes
	Pteropus niger	No	48	-	Mauritius	Joffrin et al. 2020	Yes
	Pteropus rodricensis	-	-				Yes
	Pteropus rufus	Yes	76	BetaCoV (Nobecovirus)	Madagascar	Razanajatovo et al. 2015	Yes
	Pteropus seychellensis	No	72	-	Mayotte, Seychelles	Joffrin et al. 2020	Yes
	Pteropus subniger	-	-				
	Pteropus voeltzkowi	-	-				Yes
Genus Casinycteris		0/3	7				
	Casinycteris argynnis	No	7	-	Cameroon, Republic of Congo	Anthony et al. 2017a	
	Casinycteris campomaanensis	-	-				
	Casinycteris ophiodon	-	-				
Genus Epomophorus		3/10	468				
	Epomophorus angolensis	-	-				
	Epomophorus anselli	-	-				
	Epomophorus crypturus	-	-				
	Epomophorus dobsonii	-	-				
	Epomophorus gambianus	Yes	158	AlphaCoV (Novel); BetaCoV (Novel; Nobecovirus)	Cameroon, Democratic Republic of the Congo, South Africa, Central African Republic, Senegal, Guinea	Anthony et al. 2017a, Geldenhuys et al. 2013, Maganga et al. 2014, Yinda et al. 2018, Lacroix et al. 2020	Yes
	Epomophorus grandis	-	-				
	Epomophorus labiatus	Yes	167	AlphaCoV (Novel); BetaCoV (Novel)	Rwanda, Kenya, Senegal, South Africa, Central African Republic, Cameroon	Tao et al. 2017; Nziza et al. 2019; Anthony et al. 2017a, Maganga et al. 2014, Geldenhuys et al. 2013	Yes
	Epomophorus minimus	-	-				
	Epomophorus minor	-	-				
	Epomophorus wahlbergi	Yes	80	BetaCoV (Nobecovirus)	Kenya, South Africa, Tanzania	Geldenhuys et al. 2013, Tong et al., 2009; Tao et al. 2017	
	not determined to species level (spp.)	No	63	-	Rwanda, Republic of Congo, Tanzania	Markotter et al. 2019, Anthony et al. 2017a	
Genus Epomops		1/2	1272				
	Epomops buettikoferi	No	3	-	Guinea	Lacroix et al. 2020	
	Epomops franqueti	Yes	1257	BetaCoV (Nobecovirus)	Cameroon, Democratic Republic of the Congo, Republic of the Congo, Gabon	Anthony et al. 2017a, Maganga et al. 2014	Yes

	not determined to species level (spp.)	No	12	-	Democratic Republic of the Congo, Republic of Congo	Anthony et al. 2017a	
Genus Hypsignathus		0/1	279				
	Hypsignathus monstrosus	No	279	-	Democratic Republic of the Congo, Republic of the Congo, Gabon, Cameroon, Guinea	Maganga et al. 2014, Anthony et al. 2017a, Lacroix et al. 2020	Yes
Genus Megaloglossus		1/2	456				
	Megaloglossus azagnyi	-	-				
	Megaloglossus woermanni	Yes	395	AlphaCoV (Novel), BetaCoV (Novel)	Republic of the Congo, Central African Republic, Gabon, Democratic Republic of the Congo, Cameroon	Anthony et al. 2017a, Maganga et al. 2014	
	not determined to species level (spp.)	No	61	-	Democratic Republic of the Congo	Anthony et al. 2017a	
Genus Micropteropus		1/2	892				
	Micropteropus intermedius	-	-				
	Micropteropus pusillus	Yes	892	AlphaCoV (Novel), BetaCoV (Novel)	Republic of the Congo, Gabon, Central African Republic, Cameroon, Democratic Republic of the Congo, Guinea	Anthony et al. 2017a, Maganga et al. 2014, Lacroix et al. 2020	Yes
Genus Myonycteris		1/5	832				
	Myonycteris angolensis	Yes	212	BetaCoV (Novel; Nobecovirus)	Rwanda, Kenya, Ghana, Cameroon, Republic of Congo, Uganda, Guinea)	Nziza et al. 2019; Anthony et al. 2017a, Corman et al. 2015, Tao et al. 2017, Tong et al., 2009, Lacroix et al. 2020	Yes
	Myonycteris brachycephala	-	-				Yes
	Myonycteris leptodon	-	-				
	Myonycteris relicta	-	-				
	Myonycteris torquata	No	616	-	Republic of the Congo, Gabon, Central African Republic, Cameroon, Democratic Republic of the Congo	Maganga et al. 2014, Anthony et al. 2017a	
	not determined to species level (spp.)	Yes	4	BetaCoV (Nobecovirus)	Democratic Republic of the Congo, Republic of Congo	Anthony et al. 2017a	
Genus Nanonycteris		1/1	6				
	Nanonycteris veldkampii	Yes	6	BetaCoV (Nobecovirus)	Cameroon, Guinea	Anthony et al. 2017a, Lacroix et al. 2020	
Genus Plerotes		0/1	4				
	Plerotes anchietae	No	4	-	Uganda	Anthony et al. 2017a	
Genus Rousettus		2/3	3315				
	Rousettus aegyptiacus	Yes	3129	AlphaCoV (Novel); BetaCoV (Nobecovirus)	Kenya, Egypt, Cameroon, Rwanda, South Africa, Gabon, Republic of the Congo, Central African Republic, Ghana, Democratic Republic of the Congo, Tanzania, Uganda, Guinea	Corman et al. 2015, Geldenhuys et al. 2013, Ithete et al. 2013, Maganga et al. 2014, Markotter et al. 2019, Tong et al. 2009, Tao et al. 2017, Anthony et al. 2017a, Maganga et al. 2020, Lacroix et al. 2020	Yes
	Rousettus madagascariensis	Yes	186	BetaCoV (Nobecovirus)	Madagascar	Razanajatovo et al. 2015, Joffrin et al. 2020	Yes
	Rousettus obliviosus	-	-				
Genus Scotonycteris		0/3	6				
	Scotonycteris bergmansi	-	-				
	Scotonycteris occidentalis	-	-				
	Scotonycteris zenkeri	No	6	-	Cameroon	Anthony et al. 2017a	
Genus Stenonycteris		0/1	22				
	Stenonycteris lanosus	No	22	-	Rwanda	Nziza et al. 2019; Anthony et al. 2017a	Yes
FAMILY Hipposideridae		8/21 (10 tested; 11 not tested)	8563				
Genus Asellia		0/3	0				
	Asellia italosomalica	-	-				
	Asellia patrizii	-	-				

	Asellia tridens	-	-				
Genus Doryrhina		0/1	0				
	Doryrhina cyclops	-	-				
Genus Hipposideros		5/13	7366				
	Hipposideros abae	Yes	620	AlphaCoV (Duvinavirus)	Ghana	Corman et al. 2015, Pfefferle et al., 2009, Annan et al. 2013	
	Hipposideros beatus	No	3	-	Cameroon, Republic of Congo	Anthony et al. 2017a,	
	Hipposideros caffer	Yes	1181	AlphaCoV (Duvinavirus); BetaCoV (Hibecovirus)	Kenya, Cameroon, South Africa, Democratic Republic of the Congo, Gabon, Republic of Congo, Rwanda, Mozambique	Waruhiu et al. 2017, Anthony et al. 2017a, Geldenhuys et al. 2013, Ithete et al. 2013, Nziza et al. 2019, Joffrin et al. 2020	
	Hipposideros camerunensis	-	-				
	Hipposideros cf. centralis	-	-				
	Hipposideros curtus	Yes	7	AlphaCoV (Duvinavirus)	Cameroon	Anthony et al. 2017a	
	Hipposideros fuliginosus	Yes	7	Unknown	Ghana, Cameroon	Annan et al. 2013, Anthony et al. 2017a	
	Hipposideros jonesi	No	31	-	Ghana	Annan et al. 2013	Yes
	Hipposideros lamottei	-	-				Yes
	Hipposideros marisae	-	-				Yes
	Hipposideros megalotis	-	-				
	Hipposideros ruber	Yes	5213	AlphaCoV (Duvinavirus); BetaCoV (Sarbecovirus, Hibecovirus)	Ghana, Gabon, Republic of Congo, Kenya, Rwanda, Cameroon, Democratic Republic of the Congo, Uganda, Guinea	Pfefferle et al. 2009, Corman et al. 2015, Maganga et al. 2014, Tong et al., 2009, Annan et al. 2013, Markotter et al. 2019, Nziza et al. 2019; Anthony et al. 2017a, Maganga et al. 2020, Lacroix et al. 2020	Yes
	Hipposideros tephrus	-	-				
	not determined to species level (spp.)	Yes	304	AlphaCoV (Duvinavirus); BetaCoV (Hibecovirus)	Zimbabwe, Kenya, Republic of Congo, Cameroon, Democratic Republic of the Congo, Gabon, Uganda	Bourgarel et al. 2018, Tao et al. 2017, Anthony et al. 2017a	
Genus Macronycteris		3/4	1197				
	Macronycteris commersoni	Yes	70	AlphaCoV (Novel)	Cameroon, Madagascar	Anthony et al. 2017a, Joffrin et al. 2020	Yes
	Macronycteris cryptovalorona	-	-				
	Macronycteris gigas	Yes	1004	AlphaCoV (Novel), BetaCoV (Hibecovirus,Nobecovirus)	Ghana, Gabon, Central African Republic, Nigeria, Cameroon, Republic of Congo	Annan et al. 2013, Maganga et al. 2014, Quan et al. 2010, Tong et al. 2009, Anthony et al. 2017a, Maganga et al. 2020	Yes
	Macronycteris vittatus	Yes	123	AlphaCoV (Duvinavirus)	Kenya	Tao et al. 2017	Yes
FAMILY Molossidae		8/44 (16 tested; 28 not tested)	2144				
Genus Chaerephon		2/15	601				
	Chaerephon aloysiisabaudiae	-	-				
	Chaerephon ansorgei	-	-				Yes
	Chaerephon atsinanana	No	29	-	Madagascar	Joffrin et al. 2020	
	Chaerephon bemmeleni	-	-				
	Chaerephon bivittatus	-	-				
	Chaerephon chapini	-	-				
	Chaerephon gallagheri	-	-				
	Chaerephon jobimena	-	-				Yes
	Chaerephon leucogaster	No	45	-	Madagascar	Joffrin et al. 2020	
	Chaerephon major	No	1	-	Cameroon	Anthony et al. 2017a	
	Chaerephon nigeriae	-	-				

	Chaerephon pumilus	Yes	355	AlphaCoV (Novel)	Kenya, South Africa, Tanzania, Republic of the Congo, Rwanda, Cameroon, Democratic Republic of the Congo, Uganda	Geldenhuys et al. 2013, Ithete et al. 2013, Tao et al. 2017, Tong et al. 2009, Waruhiu et al. 2017, Anthony et al. 2017a, Nziza et al. 2019	
	Chaerephon pusillus	Yes	60	AlphaCoV (Novel)	Mayotte	Joffrin et al. 2020	
	Chaerephon russatus	-	-				
	Chaerephon tomensis	-	-				Yes
	not determined to species level (spp.)	Yes	171	AlphaCoV (Novel); BetaCoV (Sarbecovirus, Nobecovirus)	Kenya, South Africa, Ghana, Democratic Republic of the Congo, Mayotte, Guinea	Tong et al. 2009, Tao et al. 2017, Pfefferle et al. 2009, Anthony et al. 2017a, Joffrin et al. 2020, Lacroix et al. 2020	
Genus Mops<br>Subgenus Mops (Mops)		2/8	1012				
	Mops (Mops) condylurus	Yes	858	AlphaCoV (Novel); BetaCoV (Novel, Nobecovirus-like)	Kenya, Cameroon, South Africa, Central African Republic, Rwanda, Democratic Republic of the Congo, Republic of Congo, Tanzania, Mozambique, Guinea	Waruhiu et al. 2017, Anthony et al. 2017a, Joffrin et al. 2020, Lacroix et al. 2020	
	Mops (Mops) congicus	-	-				
	Mops (Mops) demonstrator	No	7	-	Cameroon	Anthony et al. 2017a	
	Mops (Mops) leucostigma	No	94	-	Madagascar	Joffrin et al. 2020	Yes
	Mops (Mops) midas	Yes	23	AlphaCoV (Novel)	South Africa; Madagascar	Geldenhuys et al. 2013, Joffrin et al. 2020	Yes
	Mops (Mops) niangarae	-	-				
	Mops (Mops) niveiventer	-	-				
	Mops (Mops) trevori	-	-				
	not determined to species level (spp.)	No	30	-	Democratic Republic of the Congo, Republic of Congo	Anthony et al. 2017a	
Subgenus Mops (Xiphonycteris)		0/6	2				
	Mops (Xiphonycteris) bakarii	-	-				
	Mops (Xiphonycteris) brachypterus	No	2	-	Democratic Republic of the Congo	Anthony et al. 2017a	
	Mops (Xiphonycteris) nanulus	-	-				
	Mops (Xiphonycteris) petersoni	-	-				
	Mops (Xiphonycteris) spurrelli	-	-				
	Mops (Xiphonycteris) thersites	-	-				
Genus Mormopterus		2/3	119				
	Mormopterus acetabulosus	No	6	-	Mauritius	Joffrin et al. 2020	
	Mormopterus francoismoutoui	Yes	50	AlphaCoV (Novel)	Reunion Island	Joffrin et al. 2020	
	Mormopterus jugularis	Yes	63	AlphaCoV (Novel)	Madagascar	Joffrin et al. 2020	Yes
Genus Myopterus		0/2	0				
	Myopterus daubentonii	-	-				
	Myopterus whitleyi	-	-				
Genus Otomops		1/3	339				
	Otomops harrisoni	-	-				
	Otomops madagascariensis	No	18	-	Madagascar	Joffrin et al. 2020	
	Otomops martiensseni	Yes	321	AlphaCoV (Novel)	Rwanda, Kenya	Markotter et al. 2019, Nziza et al. 2019, Tong et al. 2009, Tao et al. 2017, Waruhiu et al. 2017, Anthony et al. 2017a	
Genus Platymops		0/3	0				
	Platymops setiger	-	-				
Genus Sauromys		0/1	0				
	Sauromys petrophilus	-	-				
Genus Tadarida		1/5	71				
	Tadarida aegyptiaca	No	7	-	South Africa	Geldenhuys et al. 2013, Ithete et al. 2013	
	Tadarida fulminans	-	-				
	Tadarida lobata	-	-				
	Tadarida teniotis	-	-				

	Tadarida ventralis	-	-				
	not determined to species level (spp.)	Yes	64	AlphaCoV (Novel); BetaCoV (Novel)	Tanzania, Uganda	Anthony et al. 2017a	
FAMILY Miniopteridae		5/22 (12 tested; 10 not tested)	1464				
Genus Miniopterus		5/22	1464				
	Miniopterus aelleni	-	-				
	Miniopterus africanus	Yes	9	AlphaCoV (Novel)	Kenya	Tong et al. 2009, Tao et al. 2017	
	Miniopterus ambohitrensis	-	-				
	Miniopterus brachytragos	-	-				
	Miniopterus egeri	-	-				
	Miniopterus fraterculus	-	-				
	Miniopterus gleni	No	16	-	Madagascar	Joffrin et al. 2020	Yes
	Miniopterus griffithsi	-	-				
	Miniopterus griveaudi	No	28	-	Madagascar	Joffrin et al. 2020	
	Miniopterus inflatus	Yes	653	AlphaCoV (Novel)	Kenya, Gabon, Cameroon, Republic of Congo	Tong et al. 2009, Tao et al. 2017, Maganga et al. 2014, Anthony et al. 2017a, Maganga et al. 2020	
	Miniopterus maghrebensis	No	7	-	Morocco	Ar Gouilh et al. 2018	
	Miniopterus mahafaliensis	No	8	-	Madagascar	Joffrin et al. 2020	
	Miniopterus majori	-	-				Yes
	Miniopterus manavi	No	19	-	Madagascar	Joffrin et al. 2020	Yes
	Miniopterus minor	Yes	311	AlphaCoV (Novel)	Kenya, Tanzania	Tong et al. 2009, Tao et al. 2017, Waruhiu et al. 2017, Anthony et al. 2017a	
	Miniopterus mossambicus	Yes	21	AlphaCoV (Novel)	Mozambique	Joffrin et al. 2020	
	Miniopterus natalensis	Yes	87	AlphaCoV (Novel)	Kenya, South Africa	Tong et al. 2009, Tao et al. 2017, Waruhiu et al. 2017, Ithete et al. 2013	
	Miniopterus newtoni	-	-				Yes
	Miniopterus petersoni	-	-				
	Miniopterus schreibersii	No	1	-	Cameroon	Anthony et al. 2017a	
	Miniopterus sororculus	No	8	-	Madagascar	Joffrin et al. 2020	
	Miniopterus villiersi	-	-				
	not determined to species level (spp.)	Yes	317	AlphaCoV (Novel)	Kenya, Democratic Republic of the Congo, Republic of Congo, Rwanda	Tao et al. 2017, Anthony et al. 2017a	
FAMILY Vespertilionidae		9/114 (37 tested; 77 not tested)	918				
Genus Kerivoula		0/7	10				
	Kerivoula africana	-	-				
	Kerivoula argentata	No	1	-	Republic of Congo	Anthony et al. 2017a	
	Kerivoula cuprosa	No	5	-			
	Kerivoula eriophora	-	-				
	Kerivoula lanosa	No	1	-	Democratic Republic of the Congo	Anthony et al. 2017a	
	Kerivoula phalaena	-	-				
	Kerivoula smithii	-	-				
	not determined to species level (spp.)	No	3	-	Cameroon, Democratic Republic of the Congo	Anthony et al. 2017a	
Genus Myotis		2/13	289				
	Myotis anjouanensis	-	-				
	Myotis bocagii	-	-				
	Myotis capaccinii	-	-				
	Myotis dieteri	-	-				
	Myotis emarginatus	-	-				
	Myotis goudoti	No	17	-	Madagascar	Joffrin et al. 2020	
	Myotis morrisi	-	-				Yes

	Myotis mystacinus	-	-				Yes
	Myotis punicus	Yes	11	AlphaCoV (Novel)	Morocco, Tunisia	Ar Gouilh et al. 2018	
	Myotis scotti	-	-				
	Myotis tricolor	-	-				
	Myotis welwitschii	Yes	257	AlphaCoV (Novel)	Rwanda, Uganda	Anthony et al. 2017a	
	Myotis zenatius	-	-				
	not determined to species level (spp.)	No	4	-	Uganda	Anthony et al. 2017a	
Genus Scotophilus		3/16	213				
	Scotophilus altilis	-	-				
	Scotophilus andrewreborii	-	-				
	Scotophilus borbonicus	-	-				
	Scotophilus dinganii	Yes	66	AlphaCoV (Novel); BetaCoV (Novel)	Kenya, Cameroon, Democratic Republic of the Congo, South Africa	Anthony et al. 2017a, Geldenhuys et al. 2013	
	Scotophilus ejetai	-	-				
	Scotophilus leucogaster	Yes	38	AlphaCoV (Novel), BetaCoV (Nobecovirus-like)	South Africa, Cameroon, Guinea	Anthony et al. 2017a, Geldenhuys et al. 2013, Lacroix et al. 2020	
	Scotophilus livingstonii	-	-				
	Scotophilus marovaza	-	-				
	Scotophilus nigrita	-	-				
	Scotophilus nigritellus	-	-				
	Scotophilus nucella	-	-				
	Scotophilus nux	Yes	3	AlphaCoV (Novel),	Cameroon	Anthony et al. 2017a	
	Scotophilus robustus	-	-				Yes
	Scotophilus tandrefana	-	-				
	Scotophilus trujilloi	-	-				
	Scotophilus viridis	No	11	-	South Africa, Rwanda, Mozambique	Nziza et al. 2019, Anthony et al. 2017a, Geldenhuys et al. 2013, Ithete et al. 2013, Joffrin et al. 2020	
	not determined to species level (spp.)	No	95	-	South Africa, Democratic Republic of the Congo	Geldenhuys et al. 2013, Anthony et al. 2017a	
Genus Barbastella		0/2	0				
	Barbastella barbastellus	-	-				
	Barbastella leucomelas	-	-				
Genus Eptesicus		0/5	4				
	Eptesicus bottae	-	-				
	Eptesicus floweri	-	-				
	Eptesicus hottentotus	No	2	-	South Africa	Geldenhuys et al. 2013	
	Eptesicus isabellinus	No	2	-	Tunisia	Ar Gouilh et al. 2018	
	Eptesicus platyops	-	-				
Genus Glauconycteris		0/13	7				
	Glauconycteris alboguttata	No	1	-	Republic of Congo	Anthony et al. 2017a	
	Glauconycteris argentata	-	-				
	Glauconycteris atra	-	-				
	Glauconycteris beatrix	No	3	-	South Africa, Ghana, Republic of Congo	Geldenhuys et al. 2013, Pfefferle et al. 2009, Anthony et al. 2017a	
	Glauconycteris curryae	-	-				
	Glauconycteris egeria	-	-				
	Glauconycteris gleni	-	-				
	Glauconycteris humeralis	-	-				
	Glauconycteris kenyacola	-	-				
	Glauconycteris machadoi	-	-				
	Glauconycteris poensis	No	2	-	Cameroon	Anthony et al. 2017a	
	Glauconycteris superba	-	-				
	Glauconycteris variegata	No	1	-	Democatic Republic of the Congo	Anthony et al. 2017a	
Genus Hypsugo		0/8	7				
	Hypsugo anchietae	-	-				
	Hypsugo ariel	-	-				
	Hypsugo bemainty	-	-				

	Hypsugo cf. eisentrauti	-	-			
	Hypsugo crassulus	No	1	-	Republic of Congo	Anthony et al. 2017a
	Hypsugo eisentrauti	-	-			
	Hypsugo musciculus	No	6	-	Cameroon	Anthony et al. 2017a
	Hypsugo savii	-	-			
Genus Laephotis		0/4	0			
	Laephotis angolensis	-	-			
	Laephotis botswanae	-	-			
	Laephotis namibensis	-	-			
	Laephotis wintoni	-	-			
Genus Mimetillus		0/2	1			
	Mimetillus moloneyi	No	1		Republic of Congo	Anthony et al. 2017a
	Mimetillus thomasi	-	-			
Genus Neoromicia		1/17	238			
	Neoromicia aff. guineensis	-	-			
	Neoromicia brunnea	No	1	-	Republic of Congo	Anthony et al. 2017a
	Neoromicia capensis	Yes	65	AlphaCoV (Novel); BetaCoV (Merbecovirus)	South Africa, Cameroon	Geldenhuys et al. 2013, Ithete et al. 2013, Geldenhuys et al. 2018, Anthony et al. 2017a
	Neoromicia guineensis	-	-			
	Neoromicia helios	No	8	-	South Africa	Geldenhuys et al. 2013, Geldenhuys et al. 2018
	Neoromicia humbloti	-	-			
	Neoromicia isabella	-	-			
	Neoromicia malagasyensis	No	2	-	Madagascar	Joffrin et al. 2020
	Neoromicia matroka	No	4	-	Madagascar	Joffrin et al. 2020
	Neoromicia nana	No	51	-	South Africa, Cameroon, Democratic Republic of the Congo, Republic of Congo, Rwanda, Tanzania, Uganda, Mozambique	Geldenhuys et al. 2013, Ithete et al. 2013, Geldenhuys et al. 2018, Anthony et al. 2017a, Joffrin etal. 2020
	Neoromicia rendalli	No	2		Democratic Republic of the Congo	Anthony et al. 2017a
	Neoromicia robertsi	-	-			
	Neoromicia roseveari	-	-			
	Neoromicia somalica	-	-			
	Neoromicia stanleyi	-	-			
	Neoromicia tenuipinnis	No	62	-	Kenya, Central African Republic, Rwanda, Cameroon, Republic of Congo	Tong et al., 2009, Tao et al. 2017, Maganga et al. 2014, Nziza et al. 2019, Anthony et al. 2017a
	Neoromicia zuluensis	No	7	-	South Africa, Rwanda	Geldenhuys et al. 2013, Geldenhuys et al. 2018, Nziza et al. 2019, Anthony et al. 2017a
	not determined to species level (spp.)	No	36	-	South Africa, Kenya	Geldenhuys et al. 2013, Tao et al. 2017
Genus Nyctalus		0/3	0			
	Nyctalus azoreum	-	-			
	Nyctalus lasiopterus	-	-			
	Nyctalus leisleri	-	-			
Genus Nycticeinops		0/1	12			
	Nycticeinops schlieffenii	No	12	-	South Africa, Cameroon, Tanzania	Geldenhuys et al. 2013, Anthony et al. 2017a
Genus Otonycteris		0/1	0			
	Otonycteris hemprichii	-	-			
Genus Pipistrellus		2/14	100			
	Pipistrellus aero	-	-			
	Pipistrellus grandidieri	-	-			
	Pipistrellus hanaki	-	-			
	Pipistrellus hesperidus	Yes	2	BetaCoV (Merbecovirus)	Uganda, Madagascar	Anthony et al. 2017b, Joffrin et al. 2020

	Pipistrellus inexspectatus	No	4	-	Cameroon	Anthony et al. 2017a	
	Pipistrellus kuhlii	Yes	32	BetaCoV (Nobecovirus-like)	Ghana, Egypt	Pfefferle et al., 2009, Shehata et al. 2016,	
	Pipistrellus maderensis	-	-				
	Pipistrellus nanulus	No	17	-	Ghana, Cameroon, Democratic Republic of the Congo, Republic of Congo	Pfefferle et al., 2009, Anthony et al. 2017a	
	Pipistrellus permixtus	-	-				
	Pipistrellus pipistrellus	-	-				
	Pipistrellus pygmaeus	-	-				
	Pipistrellus raceyi	-	-				
	Pipistrellus rueppellii	-	-				
	Pipistrellus rusticus	No	2	-	Cameroon	Anthony et al. 2017a	
	not determined to species level (spp.)	No	43	-	Kenya, Democratic Republic of the Congo, Republic of Congo	Tong et al. 2009, Tao et al. 2017, Anthony et al. 2017a	
Genus Plecotus		0/4	0				
	Plecotus balensis	-	-				
	Plecotus christii	-	-				
	Plecotus gaisleri	-	-				
	Plecotus teneriffae	-	-				
Genus Scotoecus		1/4	37				
	Scotoecus albigula	No	8	-	Uganda	Anthony et al. 2017a	
	Scotoecus albofuscus	No	3	-	Uganda	Anthony et al. 2017a	
	Scotoecus hindei	-	-				
	Scotoecus hirundo	No	1	-	Cameroon	Anthony et al. 2017a	
	not determined to species level (spp.)	Yes	25	AlphaCoV (Novel)	Kenya, Tanzania	Tao et al. 2017, Anthony et al. 2017a	
FAMILY Rhinolophidae		9/38 (14 tested; 24 not tested)	728				
Genus Rhinolophus		9/38	728				
	Rhinolophus adami	-	-				
	Rhinolophus alcyone	Yes	25	AlphaCoV (Novel)	Ghana, Gabon, Cameroon	Annan et al. 2013, Maganga et al. 2014, Anthony eta l. 2017a	Yes
	Rhinolophus blasii	-	-				
	Rhinolophus capensis	No	1	-	South Africa	Geldenhuys et al. 2013	
	Rhinolophus clivosus	Yes	79	BetaCoV (Sarbecovirus; Hibecovirus)	Rwanda, South Africa, Uganda	Markotter et al. 2019; Nziza et al. 2019; Anthony et al. 2017a,Ithete et al. 2013	
	Rhinolophus cohenae	-	-				
	Rhinolophus damarensis	-	-				
	Rhinolophus darlingi	Yes	8	AlphaCoV (Novel)	South Africa, Guinea	Geldenhuys et al. 2013, Ithete et al. 2013, Lacroix et al. 2020	
	Rhinolophus deckenii	-	-				
	Rhinolophus denti	No	5	-	South Africa	Geldenhuys et al. 2013	
	Rhinolophus eloquens	No	2	-	Rwanda	Anthony et al. 2017a	
	Rhinolophus euryale	Yes	3	AlphaCoV (Novel)	Morocco	Ar Gouilh et al. 2018	
	Rhinolophus ferrumequinum	No	2	-	Morocco	Ar Gouilh et al. 2018	
	Rhinolophus fumigatus	Yes	2	AlphaCoV (Novel)	Kenya, Cameroon	Waruhiu et al. 2017, Anthony et al. 2017a	
	Rhinolophus gorongosae	-	-				
	Rhinolophus guineensis	-	-				Yes
	Rhinolophus hildebrandtii	Yes	20	AlphaCoV (Novel); BetaCoV (Sarbecovirus)	Kenya	Tao et al. 2017, Tong et al. 2009	
	Rhinolophus hilli	-	-				Yes
	Rhinolophus hillorum	-	-				Yes
	Rhinolophus hipposideros	-	-				
	Rhinolophus horaceki	-	-				
	Rhinolophus kahuzi	-	-				

	Rhinolophus landeri	Yes	92	AlphaCoV (Novel)	Kenya, Ghana, South Africa, Cameroon	Tao et al. 2017; Waruhiu et al. 2017, Geldenhuys et al. 2013, Annan et al. 2013, Anthony et al. 2017a	
	Rhinolophus lobatus	Yes	9	AlphaCoV (Novel)	Mozambique	Joffrin et al. 2020	
	Rhinolophus mabuensis	-	-				
	Rhinolophus maclaudi	-	-				Yes
	Rhinolophus maendeleo	-	-				
	Rhinolophus mehelyi	-	-				
	Rhinolophus mossambicus	No	20	-	Mozambique	Joffrin et al. 2020	
	Rhinolophus rhodesiae	Yes	30	AlphaCoV (Novel)	Mozambique	Joffrin et al. 2020	
	Rhinolophus ruwenzorii	-	-				Yes
	Rhinolophus sakejiensis	-	-				
	Rhinolophus silvestris	-	-				Yes
	Rhinolophus simulator	-	-				
	Rhinolophus smithersi	-	-				
	Rhinolophus swinnyi	-	-				
	Rhinolophus willardi	-	-				
	Rhinolophus ziama	-	-				Yes
	not determined to species level (spp.)	Yes	430	AlphaCoV (Novel); BetaCoV (Sarbecovirus)	Kenya, Rwanda, South Africa, Democratic Republic of the Congo, Gabon, Republic of Congo, Uganda, Mozambique	Tao et al. 2017, Tao et al. 2019, Anthony et al. 2017a, Tong et al. 2009, Geldenhuys et al. 2013, Joffrin et al. 2020	
FAMILY Emballonuridae		0/11 (4 tested; 7 not tested)	678				
Genus Coleura		0/3	550				
	Coleura afra	No	550	-	Kenya, Ghana, Gabon, Cameroon, Tanzania, Uganda	Tong et al., 2009; Tao et al. 2017, Pfefferle et al., 2009; Annan et al. 2013, Corman et al. 2015, Maganga et al. 2014, Anthony et al. 2017a, Maganga et al. 2020	
	Coleura kibomalandy	-	-				
	Coleura seychellensis	-	-				
Genus Paremballonura		0/2	0				
	Paremballonura atrata	-	-				Yes
	Paremballonura tiavato	-	-				
Genus Saccolaimus		0/1	0				
	Saccolaimus peli	-	-				
Genus Taphozous		0/5	128				
	Taphozous hamiltoni	-	-				
	Taphozous hildegardeae	No	3	-	Kenya	Tong et al., 2009;	
	Taphozous mauritianus	No	19	-	Cameroon, Democratic Republic of the Congo, Tanzania, Mauritius	Anthony et al. 2017a, Joffrin et al. 2020	Yes
	Taphozous nudiventris	-	-				
	Taphozous perforatus	No	103	-	Ghana, Egypt	Annan et al. 2013, Shehata et al. 2016	
	not determined to species level (spp.)	No	3	-	Kenya, Tanzania	Tong et al., 2009, Anthony et al. 2017a	
FAMILY Nycteridae		3/15 (6 tested; 9 not tested)	299				
Genus Nycteris		2/15	299				
	Nycteris arge	-	3	-	Uganda	Anthony et al. 2017a	
	Nycteris aurita	-	-				
	Nycteris cf. parisii	-	-				
	Nycteris gambiensis	Yes	185	BetaCoV (Merbecovirus)	Ghana	Annan et al. 2013	
	Nycteris grandis	No	26	-	Cameroon	Anthony et al. 2017a	Yes

	Nycteris hispida	No	34	-	Ghana, Rwanda, Cameroon, Republic of Congo	Pfefferle et al., 2009, Nziza et al. 2019, Anthony et al. 2017a	
	Nycteris intermedia	-	-				
	Nycteris macrotis	Yes	3	BetaCoV (Merbecovirus)	Guinea	Lacroix et al. 2020	
	Nycteris madagascariensis	-	-				
	Nycteris major	No	1	-	Cameroon	Anthony et al. 2017a	
	Nycteris nana	-	-				
	Nycteris parisii	-	-				
	Nycteris thebaica	Yes	19	BetaCoV (Merbecovirus)	South Africa, Cameroon, Mozambique	Geldenhuys et al. 2013, Ithete et al. 2013, Anthony et al. 2017a, Joffrin et al. 2020	
	Nycteris vinsoni	-	-				
	Nycteris woodi	-	-				
	not determined to species level (spp.)	No	28	-	Kenya, Democratic Republic of the Congo, Republic of Congo	Tao et al. 2017, Anthony et al. 2017a	
FAMILY Rhinonycteridae		2/6 (3 tested; 3 not tested)	250				
Genus Cloeotis		0/1	0				
	Cloeotis percivali	-	-				
Genus Paratriaenops		0/3	32				
	Paratriaenops auritus	-	-				
	Paratriaenops furculus	No	32	-	Madagascar	Joffrin et al. 2020	Yes
	Paratriaenops pauliani	-	-				
Genus Triaenops		2/2	218				
	Triaenops afer	Yes	184	AlphaCoV (Setracovirus); BetaCoV (Novel)	Kenya, Republic of the Congo, Tanzania, Mozambique	Tao et al. 2017, Anthony et al. 2017a; Joffrin et al. 2020	
	Triaenops menamena	Yes	34	AlphaCoV (Setracovirus);	Madagascar	Joffrin et al. 2020	Yes
FAMILY Megadermatidae		1/2 (2 tested; 0 not tested)	25				
Genus Cardioderma		1/1	21				
	Cardioderma cor	Yes	21	AlphaCoV (Novel)	Kenya	Tao et al. 2017, Tong et al. 2009	
Genus Lavia		0/1	4				
	Lavia frons	No	4	-	Rwanda, Cameroon	Nziza et al. 2019; Anthony et al. 2017a	
FAMILY Rhinopomatidae		0/3 (1 tested, 2 not tested)	1				
Genus Rhinopoma		0/3	1				
	Rhinopoma cystops	-	-				
	Rhinopoma macinnesi	-	-				
	Rhinopoma microphyllum	No	1	-	Cameroon	Anthony et al. 2017a	
FAMILY Myzopodidae		0/2 (0 tested, 2 not tested)	0				
Genus Myzopoda		0/2	0				
	Myzopoda aurita	-	-				Yes
	Myzopoda schliemanni	-	-				
FAMILY Cistugonidae		0/2 (0 tested; 2 not tested)	0				
Genus Cistugo		0/2	0				
	Cistugo lesueuri	-	-				
	Cistugo seabrae	-	-				

Table S5: Bat species from which coronavirus RNA have been reported (according to viruses identified)

Genus	Virus subgenus/clade possibly belonging to*	Relatedness/ greatest similarity to known species	Bat species	Country	Sample type	Number tested (percentage positive)	Reference
	Duvinacovirus (HumanCoV-229E and related sequences from bat and camelids)	*Similar to HumanCoV-229E*	*Hipposideros abae*	Ghana	Fecal	19/242 (7.85%)	Corman et al. 2015
			Hipposideros caffer	Kenya	Fecal	4#	Waruhiu et al. 2017
				Cameroon	Rectal swab	1/167 (0.6%)	Anthony et al. 2017a/ PREDICT1&2*
				Gabon	Rectal (& oral) swabs	3/859 (0.3%)	Anthony et al. 2017a/ PREDICT1&2*
				COG	Rectal (& oral) swabs	1/1 (100%)	Anthony et al. 2017a/ PREDICT1&2*
				Rwanda	Rectal (& oral) swabs	1/65 (1.5%)	Anthony et al. 2017a/ PREDICT1&2*
				Mozambique	Rectal swab	10/59 (16.9%)	Joffrin et al. 2020
			Hipposideros curtus	Cameroon	Rectal (& oral) swabs	1/7 (14.3%)	Anthony et al. 2017a/ PREDICT1&2*
			Hipposideros ruber	Ghana	Fecal	5/59 (8.47%)	Pfefferle et al. 2009
				Ghana	Fecal	62/1611 (3.85%)	Corman et al. 2015
				Gabon	Intestine	2/387 (0.51%)	Maganga et al. 2014
				Gabon	Intestine	12/262 (5%)	Maganga et al. 2020
				COG	Rectal swab	1/8 (12.5%)	Anthony et al. 2017a/ PREDICT1&2*
				Cameroon	Rectal (& oral) swabs	37/674 (5.5%)	Anthony et al. 2017a/ PREDICT1&2*
				Uganda	Rectal (& oral) swabs	1/4 (25%)	Anthony et al. 2017a/ PREDICT1&2*
				Guinea	Rectal and oral swabs; Fecal	5/21 (23.8%)	Lecroix et al. 2020
			Hipposideros sp.	Zimbabwe	Colony-collected fecal samples	7/123 (5.7%)	Bourgarel et al. 2018
				Kenya	Fecal swabs	16/68 (23.53%)	Tao et al. 2017
				COG	Rectal swab	1/9 (11.1%)	Anthony et al. 2017a/ PREDICT1&2*
				Cameroon	Rectal (& oral) swabs	1/2 (50%)	Anthony et al. 2017a/ PREDICT1&2*
				Uganda	Rectal (& oral) swabs	1/53 (1.9%)	Anthony et al. 2017a/ PREDICT1&2*
			Macronycteris vittatus (Hipposideros vitattus)	Kenya	Fecal swabs	1/123 (0.81%)	Tao et al. 2017
			Macronycteris gigas (Hipposideros gigas)	Gabon	Intestine	1/156 (0.64%)	Maganga et al. 2020
			Rhinolophus clivosus	Uganda	Rectal (& oral) swabs	1/39 (2.6%)	Anthony et al. 2017a/ PREDICT1&2*
	Setracovirus (HumanCoV-NL63 and related sequences from bats)	*Similar to HumanCoV-NL63*	*Triaenops afer*	Kenya	Fecal swabs	8/30 (26.67%)	Tao et al. 2017
				Mozambique	Rectal swabs	9/51 (17.6%)	Joffrin et al. 2020
			Triaenops menamena	Madagascar	Intestine and rectal swabs	4/34 ()	Joffrin et al. 2020
			Triaenops sp.	COG	Rectal swabs	6/53 (11.3%)	Anthony et al. 2017a/ PREDICT1&2*
				Tanzania	Rectal swabs	42/50 (84%)	Anthony et al. 2017a/ PREDICT1&2*
			Mops condylurus	Tanzania	Rectal swabs	1/220 (0.5%)	Anthony et al. 2017a/ PREDICT1&2*
			Cardioderma cor	Kenya	Fecal swabs	1/13 (7.7%)	Anthony et al. 2017a/ PREDICT1&2*
				Kenya	Fecal swabs	2/8 (25%)	Tao et al. 2017
			Chaerephon pumilus	Kenya	Fecal swabs	2/7 (28.6%)	Tong et al. 2009
				Kenya	Fecal	5#	Waruhiu et al. 2017
				Tanzania	Rectal (& oral) swabs	42/211 (19.9%)	Anthony et al. 2017a/ PREDICT1&2*
				COG	Rectal (& oral) swabs	5/62 (8.1%)	Anthony et al. 2017a/ PREDICT1&2*
				Rwanda	Rectal swab	1/11 (9.09%) | 1/19 (5.3%)	Nziza et al. 2019 | Anthony et al. 2017a/ PREDICT1&2*
			Chaerephon pusillus	Mayotte	Rectal swab	7/60 (11.7%)	Joffrin et al. 2020
			Chaerephon sp.	Kenya	Fecal swabs	Unspecified - up to 5/38	Tong et al. 2009
				Kenya	Fecal swabs	12/113 (10.6%)	Tao et al. 2017
				DRC	Rectal (& oral) swabs	2/6 (33.3%)	Anthony et al. 2017a/ PREDICT1&2*
				Mayotte	Feces	1/4 (25%)	Joffrin et al. 2020
			Eidolon helvum	Cameroon	Plasma	2/302 (0.7%)	Anthony et al. 2017a/ PREDICT1&2*
			Epomops franqueti	Cameroon	Rectal (& oral) swabs	1/241 (0.4%)	Anthony et al. 2017a/ PREDICT1&2*
				COG	Rectal (& oral) swabs	1/133 (0.8%)	Anthony et al. 2017a/ PREDICT1&2*
			Epomophorus labiatus	Kenya	Fecal swabs	Unspecified - up to 9/35	Tao et al. 2017
			Epomophorus gambianus	Cameroon	Rectal (& oral) swabs	5/32 (15.6%)	Anthony et al. 2017a/ PREDICT1&2*
			Hipposideros commersoni (Macronycteris vittatus / M. gigas)	Cameroon	Rectal (& oral) swabs	2/51 (3.9%)	Anthony et al. 2017a/ PREDICT1&2*
			Hipposideros gigas (Macronycteris gigas)	Cameroon	Rectal (& oral) swabs	13/112 (11.6%)	Anthony et al. 2017a/ PREDICT1&2*
				COG	Rectal (& oral) swabs	1/3 (33.3%)	Anthony et al. 2017a/ PREDICT1&2*
avirus			*Micropteropus pusillus*	Cameroon	Liver, spleen	1/137 (0.7%)	Anthony et al. 2017a/ PREDICT1&2*

Alphacorona	Novel/unclassified alphacoronaviruses	*Miniopterus africanus*	Kenya	Fecal swabs	1/8 (12.5%)	Tong et al. 2009
		Miniopterus inflatus	Kenya	Fecal swabs	7/12 (58.3%)	Tong et al. 2009
			Kenya	Fecal swabs	1/2 (50%)	Tao et al. 2017
			COG	Rectal (& oral) swabs	5/6 (83.3%)	Anthony et al. 2017a/ PREDICT1&2*
		Miniopterus minor	Kenya	Fecal swabs	1/13 (7.7%)	Tong et al. 2009
			Kenya	Fecal swabs	66/292 (22.6%)	Tao et al. 2017
			Kenya	Fecal	5[#]	Waruhiu et al. 2017
		Miniopterus mossambicus	Mozambique	Rectal swabs	4/21 (19%)	Joffrin et al. 2020
		Miniopterus natalensis	South Africa	Fecal/ rectal specimens	1/14 (7.14%)	Geldenhuys et al. 2013
			Kenya	Fecal swabs	1/7 (14.3%)	Tong et al. 2009
			Kenya	Fecal swabs	9/53 (17%)	Tao et al. 2017
		Miniopterus sp.	Kenya	Fecal swabs	18/300 (6%)	Tao et al. 2017
			Rwanda	Rectal (& oral) swabs	1/1 (100%)	Anthony et al. 2017a/ PREDICT1&2*
		Megaloglossus woermanni	Cameroon	Liver, rectal (& oral) swabs	2/184 (1.1%)	Anthony et al. 2017a/ PREDICT1&2*
		Myonycteris angolensis	Guinea	Rectal swabs	1/42 (2.4%)	Lecroix et al. 2020
		Mops condylurus	Kenya	Fecal	3[#]	Waruhiu et al. 2017
			Cameroon	Rectal (& oral) swabs	16/176 (9.6%)	Nziza et al. 2019 Anthony et al. 2017a
			DRC	Rectal (& oral) swabs	7/104 (6.7%)	Anthony et al. 2017a/ PREDICT1&2*
			Tanzania	Rectal (& oral) swabs	105/220 (47.7%)	Anthony et al. 2017a/ PREDICT1&2*
			Mozambique	Rectal swabs	11/54 (20.4%)	Joffrin et al. 2020
		Mops midas	South Africa	Fecal/ rectal specimens	1/2 (50%)	Geldenhuys et al. 2013
			Madagascar	Intestine and rectal swabs	4/21 (19%)	Joffrin et al. 2020
		Mormopterus acetabulosus	Reunion Island	Rectal swabs	2/50 (4%)	Joffrin et al. 2020
		Mormopterus jugularis	Madagascar	Intestine and rectal swabs	10/63 (15.9%)	Joffrin et al. 2020
		Myotis punicus	Tunisia	Fecal	3/8 (37.5%)	Ar Gouilh et al. 2018
		Myotis welwitschii	Uganda	Rectal and oral swabs	2/256 (0.8%)	Anthony et al. 2017a/ PREDICT1&2*
		Neoromicia capensis	South Africa	Fecal/ rectal specimens	1/10 (10%)	Geldenhuys et al. 2013
			South Africa	Fecal	4/11 (36.4%)	Ithete et al. 2013
			South Africa	Fecal/ rectal specimens	1/41 (2.44%)	Geldenhuys et al. 2018
		Otomops martinsseni	Kenya	Fecal swabs	2/19 (10.5%)	Tong et al. 2009
			Kenya	Fecal swabs	10/35 (28.6%)	Tao et al. 2017
			Kenya	Fecal	22/150 (14.7%)	Waruhiu et al. 2017
			Rwanda	Rectal (& oral) swabs	5/102 (4.9%)	Anthony et al. 2017a/ PREDICT1&2*
		Pipistrellus inexspectatus	Cameroon	Rectal (& oral) swabs	2/4 (50%)	Anthony et al. 2017a/ PREDICT1&2*
		Rhinolophus alcyone	Cameroon	Rectal (& oral) swabs	1/6 (16.7%)	Anthony et al. 2017a/ PREDICT1&2*
		Rhinolophus clivosus	Uganda	Rectal (& oral) swabs	3/39 (7.7%)	Anthony et al. 2017a/ PREDICT1&2*
		Rhinolophus darlingi	Guinea	Fecal	2/5 (40%)	Lecroix et al. 2020
		Rhinolophus euryale	Morocco	Fecal	1/3 (33.3%)	Ar Gouilh et al. 2018
		Rhinolophus hildebrandtii	Kenya	Fecal swabs	Unspecified - up to 5/16	Tao et al. 2017
		Rhinolophus landeri	Kenya	Fecal swabs	7/58 (12.1%)	Tao et al. 2017
			Kenya	Fecal	1[#]	Waruhiu et al. 2017
		Rhinolophus lobatus	Mozambique	Rectal swabs	6/9 (66.7%)	Joffrin et al. 2020
		Rhinolophus fumigatus	Kenya	Fecal	1[#]	Waruhiu et al. 2017
		Rhinolophus rhodesiae	Mozambique	Rectal swabs	9/30 (30%)	Joffrin et al. 2020
		Rhinolophus sp.	Kenya	Fecal swabs	13/45 (28.9%)	Tao et al. 2017
			Rwanda	Rectal swabs	1/23 (4.3%)	Anthony et al. 2017a/ PREDICT1&2*
			Mozambique	Rectal swabs	1/2 (50%)	Joffrin et al. 2020
		Rousettus aegyptiacus	Kenya	Fecal swabs	Unspecified - up to 10/20	Tong et al. 2009
			Kenya	Fecal swabs	Unspecified - up to 18/397	Tao et al. 2017
			Tanzania	Fecal	1/453 (0.2%)	Anthony et al. 2017a/ PREDICT1&2*
			Guinea	Rectal and oral swabs	7/120 (5.8%)	Lecroix et al. 2020
		Scotoecus sp.	Tanzania	Fecal	1/1 (100%)	Anthony et al. 2017a/ PREDICT1&2*
		Scotophilus dingani	Kenya	Fecal swabs	1/14 (7.14%)	Tao et al. 2017
			Cameroon	Rectal (& oral) swabs	1/12 (8.3%)	Anthony et al. 2017a/ PREDICT1&2*
			DRC	Rectal (& oral) swabs	1/31 (3.2%)	Anthony et al. 2017a/ PREDICT1&2*
		Scotophilus leucogaster	Cameroon	Rectal (& oral) swabs	13/33 (39.4%)	Anthony et al. 2017a/ PREDICT1&2*
		Scotophilus nux	Cameroon	Rectal swabs	2/3 (66.7%)	Anthony et al. 2017a/ PREDICT1&2*
			Chaerephon sp.	Kenya	Fecal swabs	1/38 (2.63%)	Tong et al. 2009
			Hipposideros caffer	Rwanda	Rectal (& oral) swabs	1/65 (1.5%)	Anthony et al. 2017a/ PREDICT1&2*

Betacoronavirus	*Sarbecovirus* subgenus (SARS-CoV and SARS-related viruses)	*Similar to Human SARS-CoV*	*Hipposideros ruber*	Rwanda	Rectal swab	1/2 (50%)	Nziza et al. 2019
				COG	Rectal (& oral) swabs	1/8 (12.5%)	Anthony et al. 2017a/ PREDICT1&2*
				Cameroon	Rectal (& oral) swabs	11/674 (1.6%)	Anthony et al. 2017a/ PREDICT1&2*
			Rhinolophus hildebrandtii	Kenya	Fecal swabs	Unspecified - up to 5/16	Tao et al. 2017
			Rhinolophus clivosus	Rwanda	Fecal	2/7 (28.6%)	Markotter et al. 2019
				Rwanda	Rectal (& oral) swabs	3/24 (12.5%)	Nziza et al. 2019, Anthony et al. 2017a/ PREDICT1&2*
				Uganda	Rectal (& oral) swabs	7/39 (17.9%)	Anthony et al. 2017a/ PREDICT1&2*
	Hibecovirus subgenus (Bat coronaviruses; sister clade to *Sarbecovirus*)	*Similar to Hibecovirus*	*Hipposideros caffer*	Kenya	Fecal material	1[#]	Waruhiu et al. 2017
				Rwanda	Rectal swab	1/16 (6.25%) | 4/65 (6.2%)	Nziza et al. 2019 | Anthony et al. 2017a/ PREDICT1&2*
				Cameroon	rectal swab	1/167 (0.6%)	Anthony et al. 2017a/ PREDICT1&2*
				Tanzania	Rectal (& oral) swabs	2/15 (13.3%)	Anthony et al. 2017a/ PREDICT1&2*
			Hipposideros commersoni (Macronycteris vitattus / M. gigas)	Nigeria	Gastrointestinal tract	1[#]	Quan et al. 2010
			Hipposideros gigas (Macronycteris gigas)	Cameroon	Oral and rectal swab	9/112 (9%)	Anthony et al. 2017a/ PREDICT1&2*
				Gabon	Intestine	3/156 (1.92%)	Maganga et al. 2020
			Hipposideros ruber	Ghana	Fecal	7/59 (11.86%)	Pfefferle et al. 2009
				Gabon	Intestine	1/387	Maganga et al. 2014
				Rwanda	Rectal swab	1/13 (7.7%)	Anthony et al. 2017a/ PREDICT1&2*
				Guinea	Rectal and oral swabs; Fecal	2/21 (9.5%)	Lecroix et al. 2020
			Hipposideros sp.	Zimbabwe	Colony-collected fecal samples	1/123 (0.81%)	Bourgarel et al. 2018
			Rhinolophus clivosus	Rwanda	Rectal swab	1/24 (4.17%)	Nziza et al. 2019 Anthony et al. 2017a
			Myonycteris angolensis (Lissonycteris angolensis)	Rwanda	Rectal swab	3/6024 (5%)	Anthony et al. 2017a/ PREDICT1&2*
	Merbecovirus subgenus (lineage C, includes MERS-related viruses aswell as more distantly related viruses)	*MERS-related species*	*Neoromicia capensis*	South Africa	Fecal	1/11 (9.09%)	Ithete et al. 2013
				South Africa	Intestine	1/41 (2.44%)	Geldenhuys et al. 2018
			Pipistrellus hesperidus	Uganda	Rectal swab	2/7 (28.2%)	Anthony et al. 2017b, PREDICT1&2*
		Novel/unclassified	*Nycteris cf. gambiensis*	Ghana	Fecal	46/185 (24.9%)	Annan et al. 2013
			Nycteris macrotis	Guinea	Fecal	1/3 (33.3%)	Lecroix et al. 2020
			Nycteris thebaica	Mozambique	Rectal swabs	4/14 (28.6%)	Joffrin et al. 2020
			Chaerephon sp.	Kenya	Fecal	Unspecified - up to 5/38	Tong et al. 2009
			Eidolon dupreanum	Madagascar	Rectal swab	1/96 (1.04%)	Razanajatovo et al. 2015
			Eidolon helvum	Kenya	Fecal swabs	6/10 (60%)	Tong et al. 2009
				Kenya	Fecal	28[#]	Waruhiu et al. 2017
				Kenya	Fecal swabs	38/181 (21%)	Tao et al. 2017
				Nigeria	Fecal	6/79 (7.6%)	Leopardi et al. 2016
				Cameroon	Fecal	13/24 pools (54%)	Yinda et al. 2018
				Tanzania	Rectal swabs	413/1432 (28.8%)	Anthony et al. 2017a/ PREDICT1&2*
				Cameroon	Rectal and oral swabs	15/302 (5%)	Anthony et al. 2017a/ PREDICT1&2*
				COG	Rectal (& oral) swabs	18/34 (52.9%)	Anthony et al. 2017a/ PREDICT1&2*
				Rwanda	Rectal swabs	13/111 (11.71%) | 31/221 (14%)	Nziza et al. 2019 | Anthony et al. 2017a/ PREDICT1&2*
				DRC	Rectal (& oral) swabs	8/69 (11.6%)	Anthony et al. 2017a/ PREDICT1&2*
				Guinea	Rectal swabs	4/9 (44.4%)	Lecroix et al. 2020
			Epomops franqueti	Cameroon	pooled spleen, liver, rectal swab	3/241 (1.2%)	Anthony et al. 2017a/ PREDICT1&2*
				DRC	Rectal (& oral) swabs	13/98 (13.3%)	Anthony et al. 2017a/ PREDICT1&2*
				COG	Rectal (& oral) swabs	14/133 (10.5%)	Anthony et al. 2017a/ PREDICT1&2*
			Epomophorus gambianus	Cameroon	Rectal (& oral) swabs	17/32 (53.1%)	Anthony et al. 2017a/ PREDICT1&2*
				Guinea	Rectal swabs and fecal	5/87 (5.7%)	Lecroix et al. 2020
			Epomophorus labiatus	Kenya	Fecal swabs	Unspecified - up to 9/35	Tao et al. 2017
				Rwanda	Rectal swab	1/97 (1.03%) | 2/94 (2.1%)	Nziza et al. 2019 | Anthony et al. 2017a/ PREDICT1&2*
			Epomophorus wahlbergi	Kenya	Fecal swabs	4/63 (6.4%)	Tao et al. 2017
			Epomophorus spp.	Tanzania	Rectal swabs	3/47 (6.4%)	Anthony et al. 2017a/ PREDICT1&2*
			Hipposideros commersoni (Macronycteris vitattus / M. gigas)	Kenya	Fecal	1/10 (10%)	Tong et al. 2009
			Megaloglossus woermanni	Cameroon	Rectal (& oral) swabs	5[#]	Anthony et al. 2017a/ PREDICT1&2*
				DRC	Rectal (& oral) swabs	8/61 (13.1%)	Anthony et al. 2017a/ PREDICT1&2*

	Nobecovirus subgenus (bat coronaviruses)	Similar to HKU9 and CMR704 species/ unclassified	*Megaloglossus woermanni*	COG	Rectal (& oral) swabs	3/95 (3.2%)	Anthony et al. 2017a/ PREDICT1&2*
				Cameroon	Rectal (& oral) swabs	7/184 (3.8%)	Anthony et al. 2017a/ PREDICT1&2*
			Micropteropus pusillus	CAF	Lung	2/533 (0.38%)	Maganga et al. 2014
				Cameroon	Rectal (& oral) swabs	7#	Anthony et al. 2017a/ PREDICT1&2*
				DRC	Pooled liver, spleen, rectal swabs	42/520 (8.1%)	Anthony et al. 2017a/ PREDICT1&2*
				COG	Rectal (& oral) swabs	5/55 (9.1%)	Anthony et al. 2017a/ PREDICT1&2*
			Mops condylurus	Cameroon	Rectal swabs	2/176 (1.2%)	Anthony et al. 2017a/ PREDICT1&2*
				COG	Rectal (& oral) swabs	1/1 (100%)	Anthony et al. 2017a/ PREDICT1&2*
				Tanzania	Rectal (& oral) swabs	1/220 (0.5%)	Anthony et al. 2017a/ PREDICT1&2*
			Myonycteris angolensis	Rwanda	Rectal swabs	1/45 (2.22%)	Nziza et al. 2019, Anthony et al. 2017a/ PREDICT1&2*
				Guinea	Rectal swabs	2/42 (4.76%)	Lecroix et al. 2020
			Myonycteris torquata	Cameroon	Rectal swabs	1/37 (2.7%)	Anthony et al. 2017a/ PREDICT1&2*
			Myonycteris spp.	DRC	Pooled liver, spleen, rectal swabs	1/1 (100%)	Anthony et al. 2017a/ PREDICT1&2*
				COG	Rectal (& oral) swabs	3/3 (100%)	Anthony et al. 2017a/ PREDICT1&2*
			Nanonycteris veldkampii	Guinea	Rectal swabs	1/1 (100%)	Lecroix et al. 2020
			Pipistrellus deserti	Egypt	Liver	1/31' (3.26%)	Shehata et al. 2016
			Pteropus rufus	Madagascar	Rectal swab	13/76 (17.1%)	Razanajatovo et al. 2015
			Rousettus aegyptiacus	Egypt	Oral, rectal, lung. Liver	17/257 (6.62%)	Shehata et al. 2016
				Kenya	Fecal	10#	Waruhiu et al. 2017
				Kenya	Fecal swabs	Unspecified - up to 10/20	Tong et al. 2009
				Kenya	Fecal swabs	Unspecified - up to 18/397	Tao et al. 2017
				Cameroon	spleen, liver, rectal swabs	9/201 (4.5%)	Anthony et al. 2017a/ PREDICT1&2*
				Rwanda	Rectal (& oral) swabs	4/36 (11.1%) | 10/286 (3.5%)	Nziza et al. 2019 | Anthony et al. 2017a/ PREDICT1&2*
				Tanzania	Rectal (& oral) swabs	20/453 (4.4%)	Anthony et al. 2017a/ PREDICT1&2*
				Guinea	Rectal and oral swabs	5/120 (4.2%)	Lecroix et al. 2020
			Rousettus madagascariensis	Madagascar	Rectal swabs	6/45 (13.3%)	Joffrin et al. 2020
			Rhinolophus sp.	DRC	Rectal (& oral) swabs	1/62 (1.6%)	Anthony et al. 2017a/ PREDICT1&2*
			Scotophilus dinganii	Cameroon	Rectal (& oral) swabs	2/12 (16.7%)	Anthony et al. 2017a/ PREDICT1&2*
			Scotophilus leucogaster	Cameroon	Rectal (& oral) swabs	2/33 (6.1%)	Anthony et al. 2017a/ PREDICT1&2*
			Triaenops sp.	Tanzania	Rectal swabs	5/50 (10%)	Anthony et al. 2017a/ PREDICT1&2*

Abbreviations: DRC - Democratic Republic of the Congo; COG - Republic of the Congo; CAF - Central African Republic

Unspecified totals tested per species - Quan et al. 2010 and Waruhiu et al. 2017

* Totals positive and numbers per species are not indicated in Anthony et al. 2017a; sample numbers were obtained from https://www.healthmap.org/predict/ as "Predict 1 & 2 surveillance and test data"

| is used to separate sample numbers indicated in Nziza et al. 2019 based on PREDICT surveillance and the totals accessed via https://www.healthmap.org/predict/ as "Predict 1 & 2 surveillance and test data"

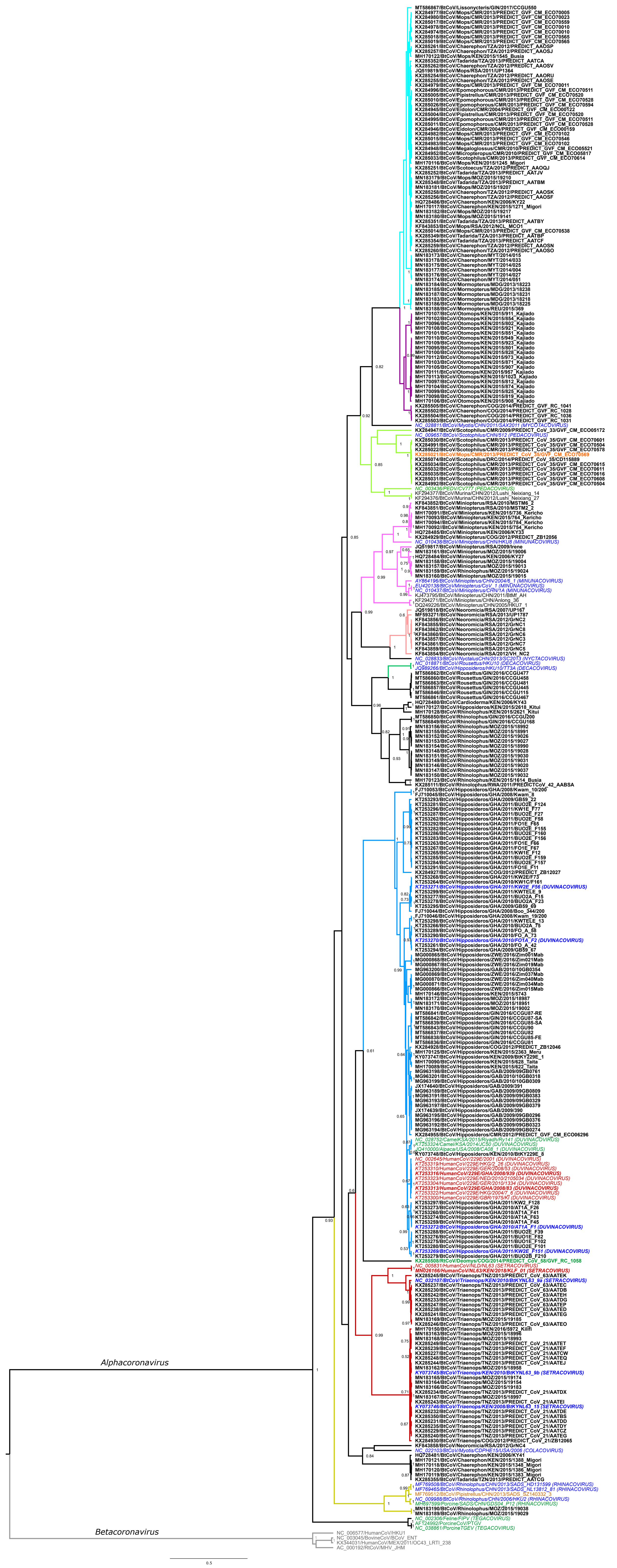

Figure S1

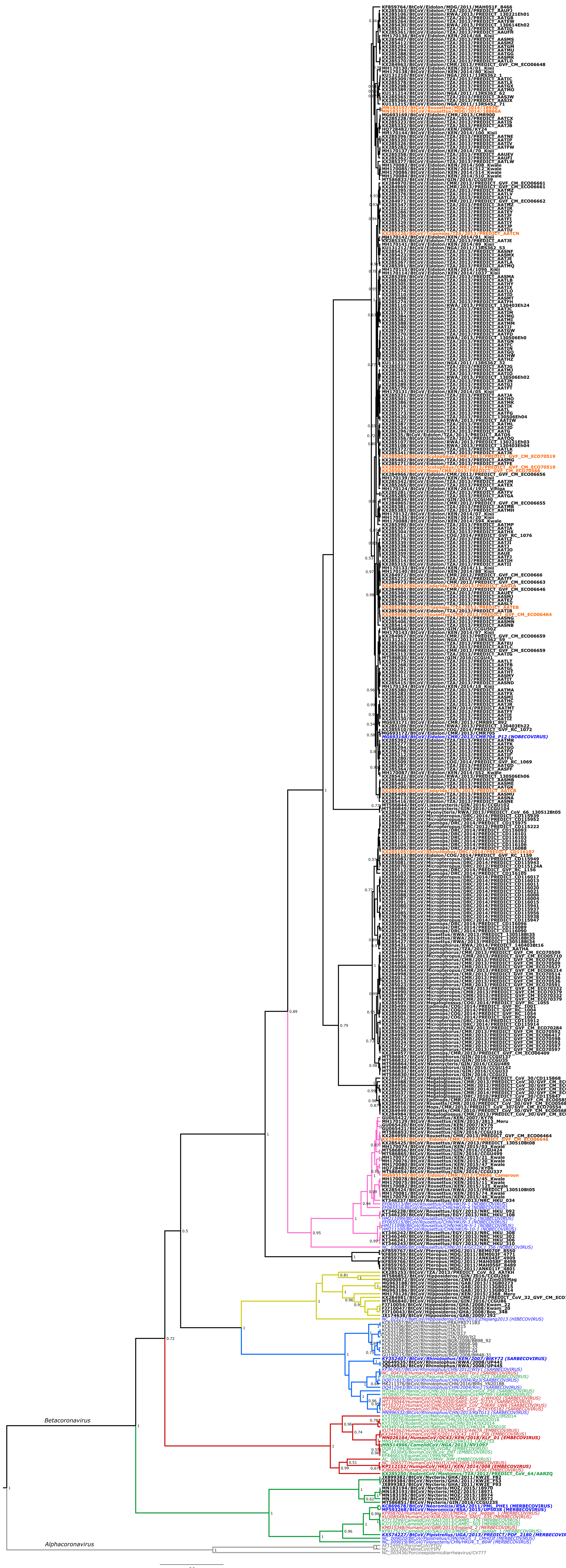

Figure S2
